# Cardiovascular diseases in the elderly: possibilities for modulating autophagy using non-coding RNAs

**DOI:** 10.3389/fcell.2025.1520850

**Published:** 2025-07-31

**Authors:** Silvia Scalabrin, Stefano Cagnin

**Affiliations:** ^1^ Department of Biology, University of Padova, Padua, Italy; ^2^ Interdepartmental Research Center of Myology (cirMYO), University of Padova, Padua, Italy

**Keywords:** cardiovascular disease, autophagy, non-coding RNAs, aging, meta-analysis

## Abstract

Autophagy is a crucial mechanism implicated in both aging and cardiovascular disease, which are two closely interconnected conditions. Modulation of autophagy is expected to have profound impacts on cellular aging and maintenance of cardiovascular functions under physiological or pathological conditions. Consequently, modulation of autophagy could be an effective strategy for counteracting age-induced vascular and cardiac remodelling as well as alleviating cardiovascular disease. The present review comprehensively elucidates the multifaceted impacts of autophagy on aging of the cardiovascular system. We comprehensively analyse both vascular and cardiac tissues, including vascular and cardiac malignancies, in distinct contexts. We also emphasize the significance of non-coding RNAs (ncRNAs) in the epigenetic regulation of gene expression and their roles as biomarkers of cardiovascular pathologies while maintaining clear distinctions between the vascular and cardiac tissues. Preclinical and clinical models are described herein to highlight the importance of ncRNAs in disease treatment by considering their involvement in the modulation of autophagy within the cardiocirculatory system. Finally, we conducted a comprehensive meta-analysis of transcriptomic data to underscore the paramount importance of autophagy while demonstrating it as a process that is frequently dysregulated in both cardiac and vascular cells under pathological conditions. The findings presented herein emphasize the importance of investigating novel strategies for modulating autophagy as a potential therapeutic approach to the management of age-related cardiovascular disorders.

## 1 Introduction

Cardiovascular disease (CVD) is a term used to indicate the range of conditions affecting the heart and blood vessels. The four main types of CVDs are coronary artery disease (CAD), strokes and transient ischemic attacks, peripheral artery disease, and aortic disease. The aorta is the primary blood vessel in the body that serves as the conduit for transporting blood from the heart to various regions of the body. One of the common problems of the aorta is an aneurysm, where the aorta becomes weakened and its wall tends to bulge outward; this region could become a site of possible rupture and cause hemorrhage that would prevent sustenance of the other parts of the body. Similar problems are known to occur in peripheral artery diseases, which are characterized by occlusion of the peripheral arteries that prevent normal blood flow. The obstruction of blood flow to specific regions of the brain can lead to cerebral infarctions, which can result in permanent brain damage or even death. Mini cerebral infarctions are defined as transient ischemic events. In both types of infarctions, the symptoms include the inability to smile, drooping of the mouth or eye, the impossibility of fitting both arms, and the inability to speak or understand communication with another person. The obstruction of oxygen-rich blood supply to the heart can also cause coronary heart diseases. Among these, it is possible to recognize angina pectoris that causes chest pain, heart attack, or heart failure. Angina is typically not life-threatening but serves as an early warning sign of the risk of a potential heart attack or stroke. In contrast, a heart attack (myocardial infarction or MI) is a serious medical emergency characterized by sudden blockage of blood supply to the heart as well as heart failure caused by the inability of the heart to pump blood to the body.

**FIGURE 1:**
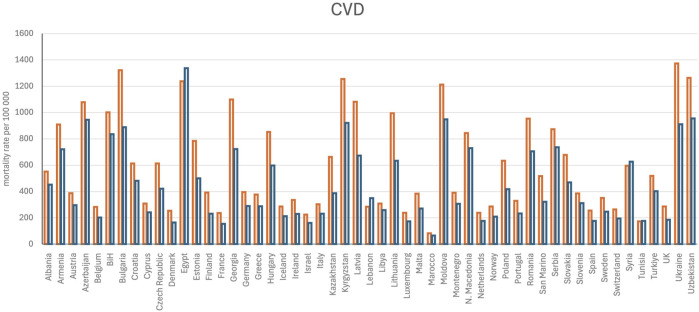
Age-standardized mortality rate per 100,000 persons for cardiovascular diseases (CVDs). The data were retrieved from Timmis et al. (2024). The orange bars represent men, and blue bars indicate women. UK, United Kingdom; BiH, Bosnia and Herzegovina.

**FIGURE 2:**
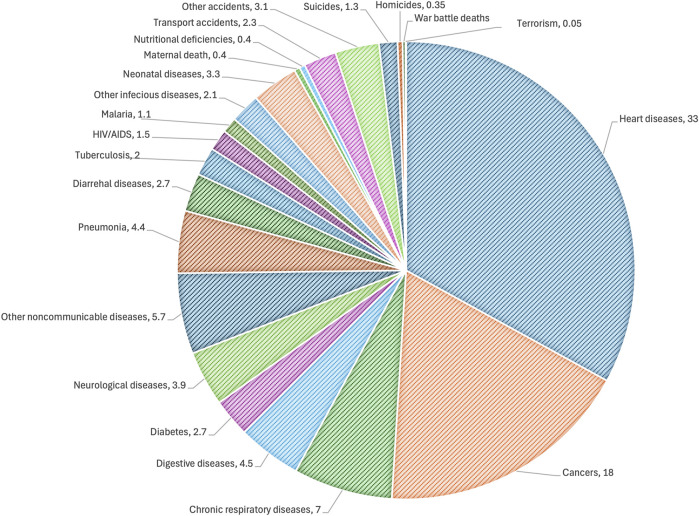
Distribution showing the global causes of death. The data were retrieved from the IHME global burden and disease and global terrorism databases. The data are representative for 2019, when the total number of deaths was 55 million.

The exact causes of CVDs are not clear, but there are several risk factors that can increase the possibility of developing cardiovascular conditions. In Europe, there were approximately 1.7 million deaths in the year 2020 from diseases of the circulatory system (343 deaths per 100,000 inhabitants; Eurostat, 2024), while the number of such deaths in the United States that had been decreasing until 2019 started increasing after the COVID-19 pandemic, reaching approximately 454.5 deaths per 100,000 inhabitants in 2022 (Woodruff et al., 2024). Interestingly, the World Health Organization reported in 2021 that the three leading causes of death were ischemic heart disease (∼9 millions), COVID-19 (slightly less than the former), and stroke (∼7 millions) (World Bank Group, 2024). Excluding the impacts of COVID-19 on death, the most prevalent pathologies responsible for mortality are associated with the cardiocirculatory system. CVD is most common in people older than 50 years, and the risk of developing CVD increases as people age further. Men are more affected than women (Figure 1) and are likely to develop CVD at an earlier age. Moreover, unhealthy dietary habits can lead to high cholesterol and high blood pressure, which are two of the important risk variables for developing CVDs. Some additional factors associated with increased risk of developing CVDs are smoking that can damage and narrow blood vessels; inactivity that can cause high blood pressure, high cholesterol, and excess bodyweight; diabetes that can damage blood vessels and cause their narrowing because of high blood sugar levels; hereditary conditions; and ethnicity (black non-Hispanic persons are the most affected in the United States) (U.S. Centers for Disease Control and Prevention, 2024). Chronic infections appear to be emerging risk factors for the development of CVDs even if they are not associated with aging. Notably, infection by the hepatitis C virus is associated with elevated incidence of atherosclerosis, strokes, and ischemic attacks (Domont and Cacoub, 2016). Chronic infection by the human immunodeficiency virus is well-known to be associated with various cardiovascular conditions, including heart failure, stroke, coronary artery plaques, and atherosclerosis (Longenecker et al., 2016; So-Armah et al., 2020; Perkins et al., 2023). Recently, there is a growing body of evidence suggesting potential associations between chronic endodontic infections and CVDs (Koletsi et al., 2021). Globally, CVDs are the most common causes of death and are more prevalent than cancers (Figure 2).

Notably, under pathological cardiac conditions, the tissues undergo substantial remodeling, necessitating turnover of the molecules and organelles akin to developmental processes. This is based on a catabolic mechanism called autophagy. Several studies have described the importance of autophagy in CVDs and the possibilities of its modulation for therapeutic interventions (Mei et al., 2014; Sciarretta et al., 2018; Jiang et al., 2023). Autophagic activities have been found to decrease with age, likely contributing to the accumulation of damaged macromolecules and organelles from aging (Barbosa et al., 2019; Aman et al., 2021). Therefore, autophagic decline occurring during aging can contribute to the development of CVDs. Non-coding RNAs (ncRNAs) have been demonstrated to play crucial roles in diverse biological processes, including aging (Dhahbi, 2016; He et al., 2018; Sherazi et al., 2023; Wagner et al., 2024a; Ugalde et al., 2024) and CVDs (Poller et al., 2017; Kohlmaier et al., 2023; Jiang and Zhang, 2024). In this work, we emphasize the interactions between mechanisms associated with aging that are related to the development of CVDs; further, we focus on coding genes that regulate autophagy and experience expression modulations related to aging as well as CVD development. Excluding the descriptions of genes associated with autophagy, we primarily consider recent publications (from the last 5 years) on aging and CVDs in this review. Moreover, we consider transcriptomic data from different CVDs (human coronary plaques, failing human heart, ischemic cardiomyopathy, and idiopathic dilated cardiomyopathy) to demonstrate the importance of autophagy modulations in these pathologies.

## 2 Approaches for investigating the relationships between CVDs and autophagy-associated ncRNAs

### 2.1 Literature search

A comprehensive literature search was performed in PubMed to select articles published from 2019 to 2024. The search terms used included the following keywords: “cardiovascular disease,” “CVD,” “heart,”, “cardiac’,” “vasculature’,” “vascular,” “aging,” “ageing,” “non-coding RNAs,” “ncRNAs,” “microRNAs,” “miRNAs,” “long non-coding RNAs,” “lncRNAs,” “circular RNAs,” “circRNAs,” and “autophagy.” The information on autophagy was sourced from articles without any restriction on the year of publication.

### 2.2 Meta-analysis based on databases

Data were downloaded from the Gene Expression Omnibus (GEO) and GEO RNA-seq Experiments Interactive Navigator (GREIN) (Mahi et al., 2019) databases. Our database search encompassed the terms “cardiovascular disease” and “cardiovascular system” and prioritized studies conducted exclusively on humans. We excluded studies involving blood and blood cells, *in vitro* studies utilizing specific cells, or studies on modulation of specific genes. In contrast to the literature search, we utilized data published between 2014 and 2024 here as the data retrieved from the past 5 years were deemed insufficient. To avoid introducing alterations that could be associated with various normalisation approaches, we opted to utilize data that were already normalized. The differentially expressed genes (DEGs) were identified using AltAnalyze software (Emig et al., 2010; Salomonis et al., 2010; Olsson et al., 2016). Genes with normalized expressions below 0.3 (no log) were removed from the analysis, and a moderate t-test with Benjamini–Hochberg correction was performed to identify the DEGs. The normalized data are shown in Supplementary Table S1. The DEGs were then categorized by an overrepresentation approach implemented in easyGSEA (Cheng et al., 2021), and the Venn diagram was obtained using Venny 2.0 (Oliveros, 2007).

### 2.3 Approaches to investigate the roles of ncRNAs in autophagy within CVDs

The ncRNAs regulate gene expressions through several mechanisms that impact the approaches used to understand their functions. The first step here is to understand if their expressions are altered under different pathological conditions. In this regard, RNA sequencing is preferred to microarray nowadays because of the similar costs and greater ability to distinguish between the long non-coding and micro RNA (lncRNA and miRNA) isoforms (isomiRs) that have demonstrated more importance (Tomasello et al., 2021). For instance, van der Kwast et al. (2020) utilized 5′Dumbbell-PCR (5′DB-PCR) to support the RNA sequencing data to demonstrate that WT-miR-411 and iso-miR-411 exhibit differential expressions between the primary human umbilical arterial fibroblasts and human umbilical venous endothelial cells (HUVECs) with different target pools. The 5′DB-PCR is based on annealing of the two stem loops at the 3′ and 5′ ends of a miRNA. Although stem loops are used as sequence bases for annealing PCR primers, the gaps or overlaps in isomiRs strongly impact the efficacy of ligation as well as annealing of the TaqMan probe partially complementary to the microRNA and partially to the 3′ adapter sequences (Tomasello et al., 2021). In particular, isomiRs remain relatively unexplored and present an open problem because miRNAs and isomiRs regulate different targets; their analyses may furnish new and alternative approaches to treating autophagy induced by ischemic events. Different isomiRs can be formed as consequences of the altered activities of RNAses type III Drosha and Dicer involved in miRNA generation, RNA editing processes, and DNA mutations (single-nucleotide polymorphisms). These are described in several databases that have collected sequencing results. Two different databases have described ncRNAs that are specifically involved in CVDs, namely, CVDncR (Wu et al., 2020) and CARDIO-LNCRNAs (Jiang et al., 2019). Databases are valuable resources for studying ncRNAs (Balamurali and Stoll, 2020; Zhang et al., 2020a), especially those that describe validated interactions, such as the Encyclopedia of RNA Interactomes (Yang et al., 2011; Li et al., 2014). Indeed, identification of ncRNA interactors would enable formulation of testable hypotheses and facilitate planning of validation experiments. Notably, ncRNAs exhibit higher cell-type-specific expression patterns than mRNAs. Therefore, their identification in individual cells is crucial. This is now feasible through various approaches based on single-cell RNA sequencing (scRNA-seq) (Jovic et al., 2022). Although scRNA-seq offers significant advantages, such as the ability to capture cellular heterogeneity, it has a critical a limitation in the form of loss of histological information. Spatial transcriptomic data may address this limitation by simultaneously capturing both the transcriptomic and spatial information while preserving the information of individual cells (Williams et al., 2022). Consequently, the study of ncRNAs in CVDs would require development of specialized databases based on scRNA-seq and spatial transcriptomic data obtained from specific tissues (e.g. heart and vascular tissues). It is crucial to acknowledge that scRNA-seq has limited sensitivity and lacks the ability to reliably detect low-abundance transcripts. This inherent limitation poses a challenge to the analysis of ncRNAs exhibiting lower expression levels than miRNAs.

## 3 Normal autophagy

Autophagy is a highly conserved cellular process in eukaryotes that involves the sequestration of cytoplasmic components into double-membrane vesicles called autophagosomes, which are then delivered to lysosomes for degradation and recycling (Yorimitsu and Klionsky, 2005; Mizushima and Klionsky, 2007). This process plays crucial roles in cellular homeostasis, adaptation to nutrient limitations, and protein turnover (Mizushima and Klionsky, 2007). Autophagy is regulated by a complex network of proteins and can be induced by various stressors, including starvation and endoplasmic reticulum stress (Ryter et al., 2013); it can also occur through bulk degradation or selective pathways targeting specific cargoes, such as aggregated proteins or dysfunctional mitochondria (Ryter et al., 2013). The process consists of several steps, namely, initiation, sequestration, transport to lysosomes, degradation, and utilization of degraded products, each of which can potentially serve different functions (Mizushima and Klionsky, 2007). Three types of autophagy have been identified in mammals, namely, macroautophagy, microautophagy, and chaperone-mediated autophagy (CMA) (Tettamanti et al., 2019). Tables 1–3 collectively summarize the proteins involved in the initial stages of autophagy, membrane shaping and autophagosome formation, and fusion of autophagosomes with lysosomes, respectively. Microautophagy differs from macroautophagy because it is not based on the formation of new membranes to isolate small pieces of cytoplasm for degradation. During microautophagy, the degradable portion (cargo) is selected by invaginations or protrusions of the membranes of the endo-lysosomal compartments. The cargos are then recognized by Atg8, Nbr1, Hsc70, or other proteins. Microautophagy can also uptake cytoplasmic materials non-selectively (see Table 4 for the proteins involved). However, CMA does not use membrane structures to select the degradable material (Kaushik and Cuervo, 2018). Here, the chaperone complex recognizes KFERQ-like pentapeptides that permit translocation of unfolded proteins to the lysosomes through pores formed by the multimers of Lamp2A (Agarraberes and Dice, 2001) (see Table 5 for the proteins involved).

**TABLE 1:** Autophagy initiation.

Macroautophagy			
Starvation-induced initiation			
Protein	Complex	Function	References
mTorc1		Inhibition of Ulk1	Fujioka et al. (2014)
Ulk1	Ulk complex	Autophagosome fusion	Fujioka et al. (2014)
Fip200		Ulk1 interacting protein	Turco et al. (2019)
Atg13		Target for TOR kinase signaling	Yamamoto et al. (2016)
Atg101		Interactor of Atg13	Fujioka et al. (2014)
Atg9		Associated with vesicles	Ren et al. (2023b)
Cargo-driven assembly			
In addition to proteins involved in the starvation-induced autophagy			
Ndp52	Sequestrosome	Receptor for ubiquitin-coated proteins	Vargas et al. (2019)
Sqstm1		Ubiquitin binding	Turco et al. (2019)
Tax1bp1		Ubiquitin-binding adapter	Turco et al. (2021)
Nbr1		Autophagy receptor for selective autophagic degradation of peroxisomes	Turco et al. (2021)
Optn		Vesicle trafficking (recruiting of Atg9 vesicles)	Yamano et al. (2020)
p62		Assembly of Ulk complex	Turco et al. (2021)

Macroautophagy induced by starvation starts with formation of the Ulk complex near the endoplasmic reticulum membrane and recruits Atg9 vesicles via interaction with the Atg13–Atg101 subcomplex. Alternatively, cargo-driven assembly commences with the intervention of adaptors such as p62, Ndp52, and Tax1bp1, which initiate assembly of the Ulk complex through interactions with FIP200; here, Atg9 vesicles are recruited by Optn.

**TABLE 2:** Membrane shaping during autophagy.

Membrane elongation			References
Beclin1	Pi3kc3 complex I	Mammalian ortholog of the yeast autophagy-related gene 6 (Atg6)	Baskaran et al. (2014)
Vps15		Class 3 phosphoinositide 3-kinase (PI3K)	Baskaran et al. (2014)
Vps34		Class 3 phosphoinositide 3-kinase (PI3K)	Baskaran et al. (2014)
Nrbf2		Association with PI3K complex I (PI3KC3-C1)	Baskaran et al. (2014)
Atg14		Determines localization of the autophagy-specific PI3-kinase complex PI3KC3-C1	Baskaran et al. (2014)
Proteins involved in starvation-induced autophagy	Ulk complex		
Atg5		In combination with autophagy protein 12 (Atg12), functions as an E1-like activating enzyme in a ubiquitin-like conjugating system	Bozic et al. (2020)
Atg12		Works in combination with Atg5	Bozic et al. (2020)
Atg16L1		Part of a large protein complex that is necessary for autophagy	Bozic et al. (2020)
Wipi2		Regulates the assembly of multiprotein complexes	Dooley et al. (2014)
Ub		Ubiquitins are involved in protein labeling for degradation	Kraft et al. (2010)
Atg8		Ubiquitin protein ligase binding activity and autophagosome assembly	Bozic et al. (2020)
Atg9	Association between the endoplasmic reticulum (ER) and the cup-shaped membrane structure (known as phagophore or isolation membrane)	Autophagosome assembly	Maeda et al. (2020)
Wipi4		Involved in autophagosome assembly downstream of Wipi2	Bakula et al. (2017)
Atg2		Lipid transfer protein involved in autophagosome assembly	Osawa et al. (2019)
Tmem41b		Involved in autophagosome assembly. Located in the ER and mitochondria-associated ER membranes	Ghanbarpour et al. (2021)
Vmp1		Transmembrane protein that plays a key regulatory role in autophagy	Ghanbarpour et al. (2021)
Dfcp1		Recruitment of proteins involved in membrane trafficking	Nähse et al. (2023)

Membrane closure and autophagosome formation			
Vps2	Escrt-III	Formation of endocytic multivesicular bodies	Takahashi et al. (2018)
Vps20		Formation of endocytic multivesicular bodies	Takahashi et al. (2018)
Vps24		Formation of endocytic multivesicular bodies	Takahashi et al. (2018)
Snf7		Formation of endocytic multivesicular bodies	Zhou et al. (2019)
Vps60		Formation of endocytic multivesicular bodies	Zhen et al. (2020)
Did2	Escrt-III	Formation of endocytic multivesicular bodies	Pfitzner et al. (2020)
Ist1		Interacts with components of endosomal sorting complexes required for transport	Pfitzner et al. (2020)
Vps4		Associated with the endosomal compartments	Mejlvang et al. (2018)

During the membrane-elongation step, the Ulk complex recruits the class III phosphatidylinositol 3-kinase complex I (PI3KC3–C1) that produces PI(3)P and further recruits its effector proteins: Dfcp1 to omegasomes; Wipi2 and Wipi4 to phagophores. Wipi4 directs Atg2 to the phagophore membrane, which then transfers phospholipids from the ER along with Atg9, Vmp1, and Tmem41b. Wipi2 recruits the Atg12–Atg5–Atg16l1 complex to promote LC3 lipidation on the phagophore membrane. ESCRT machinery is then involved in autophagosome closing.

**TABLE 3:** Formation of autolysosomes and endosomal recycling.

Lysosome fusion and formation of autolysosome			References
Rab7		RAS-related GTP-binding proteins that are important regulators of vesicular transport	Wang et al. (2016)
Epg5		Involved in autophagy	Wang et al. (2016)
Plekhm1		Acts as a multivalent adapter protein to regulate Rab7-dependent fusion events	McEwan et al. (2015)
Vamp7	SNARE complex	Involved in targeting and/or fusion of transport vesicles to their target membranes	Bas et al. (2018)
Vamp8		VAMP8 is a SNARE involved in autophagy through direct control of autophagosome membrane fusion with the lysosome membrane	Bas et al. (2018)
Snap29		Snap29 is a SNARE involved in autophagy	Bas et al. (2018)
Stx17		Stx17 is a SNARE involved in autophagy	Itakura et al. (2012)
Stx7		Mediates endocytic trafficking from early to late endosomes and lysosomes	Bas et al. (2018)
Ykt6		Mediates vesicle docking and fusion to a specific acceptor cellular compartment	Matsui et al. (2018)
Recycling			
Stx17		Stx17 is a SNARE involved in autophagy	Itakura et al. (2012)
Atg9		Associated with vesicles	Zhou et al. (2022)
Snx17		Critical regulator of endosomal recycling	Zhou et al. (2022)
Snx4		Involved in autophagosome assembly by regulating trafficking and recycling of phospholipid scramblase ATG9A	Zhou et al. (2022)
Snx5		Involved in several stages of intracellular trafficking	Zhou et al. (2022)
Kif5b		Microtubule-dependent motor required for normal distribution of mitochondria and lysosomes	Du et al. (2016)
Dnm2		Catalyzes the hydrolysis of GTP and utilizes this energy to mediate vesicle scission	Du et al. (2016)
Pip5k1b		Catalyses the phosphorylation of phosphatidylinositol 4-phosphate (PtdIns(4)P/PI4P) to form phosphatidylinositol 4,5-bisphosphate (PtdIns(4,5)P2/PIP2), a lipid second messenger that regulates several cellular processes like signal transduction, vesicle trafficking, actin cytoskeleton dynamics, cell adhesion, and cell motility	Rong et al. (2012)

Subsequent to autophagosome closing, lysosomes are tethered to the autophagosomes by Plekhm1, Epg5, and Rab7, while the two SNARE complexes Stx17–Snap29–Vamp7/8 and Ykt6–Snap29–Stx7 trigger fusion. Lysosomal membrane proteins on autolysosomes are recycled via autophagic lysosome reformation, whereas autophagosomal membrane proteins are recycled via autophagosomal component recycling.

**TABLE 4:** Microautophagy.

Microautophagy		References
Atg30	Key player in the selection of peroxisomes as cargo and delivery to autophagy for pexophagy	Farré et al. (2013)
Atg39	Autophagy of perinuclear ER/nucleus under nitrogen deprivation	Otto and Thumm (2021)
Sec62	Intervenes during recovery from ER stress	Loi et al. (2019)
Hsc70	Cargo recognition	Sahu et al. (2011)
cGAS	Cyclic GMP-AMP synthase: cytosolic DNA sensor	Zhao et al. (2021b)

Microautophagy is regulated by the invagination of endosomal or lysosomal membranes to incorporate cytoplasmic material. It can be recognized from Atg8, Nbr1, Ub (proteins shared with macroautophagy as noted in the table), Atg30, Atg39, Sec62, cGAS, Hsc70, and Tsg101.

**TABLE 5:** List of proteins involved in chaperone-mediated autophagy.

Chaperone-mediated autophagy		
Protein	Function	References
Hsc70 (also known as Hspa8)	Cytoplasmic Hsc70: recognition of KFERQ-like motif binding to lysosome-associated membrane protein (LAMP2A) Lysosomal Hsc70: substrate translocation in the lysosome	Kaushik and Cuervo (2018)
Hsp40 or Dnabj1	Co-chaperones that participate in substrate unfolding when bound to Hsc70 and membrane	Kaushik and Cuervo (2018)
HOP (Hsp70–Hsp90 organizing protein)		Kaushik and Cuervo (2018)
Hsp90		Bandyopadhyay et al. (2008)
HIP (Hsp70-interacting protein)		Kaushik and Cuervo (2018)
Cathepsins	Protein degradation within the lysosome	Kaminskyy and Zhivotovsky (2012)
Lamp2A	Receptor for internalization in the lysosomes of targeted proteins	Qiao et al. (2023)
GFAP	Filament protein involved in modulation of stability of Lamp2A	Mehrbod et al. (2019)
Ef1a	Partner of GFAP	Kaushik and Cuervo (2018)

The process starts with recognition of the KFERQ-like motif in the proteins that are degraded by Hsc70 and co-chaperones. This complex is guided to the Lamp2A receptor. Lamp2A is formed on the surfaces of the lysosomes through GFAP. The lysosome component of Hsc70 allows the protein to be degraded to enter the lysosome where it is degraded by cathepsins.

## 4 Autophagy in aging and age-related CVDs

### 4.1 Autophagy in aging

Aging is the most important risk factor for age-related diseases, such as neurodegenerative diseases, CVDs, metabolic diseases, musculoskeletal diseases, and diseases of the immune system (Kubben and Misteli, 2017; Guo et al., 2022). Many elderly people have multiple comorbidities with advancing age (Guo et al., 2022). López-Otín et al. (2023) have suggested twelve molecular, cellular, and systemic hallmarks of aging as follows: DNA instability, telomere attrition, epigenetic alterations, loss of proteostasis, disabled macroautophagy, deregulated nutrient-sensing mechanisms, mitochondrial dysfunction, cellular senescence, stem-cell exhaustion, altered intercellular communication, chronic inflammation, and dysbiosis. These hallmarks are interdependent, meaning that the experimental accentuation or attenuation of a specific hallmark can affect the others as well (Guo et al., 2022). Impairment of autophagy was proposed for the first time as a hallmark of aging by Aman et al. (2021). There is increasing evidence that autophagy-related gene expressions and autophagic activities decrease with age in different tissues in different species. Forced genetic impairment of autophagy has been reported to accelerate the decline of cellular functions (Guo et al., 2022), where tissue-specific knockout of the autophagy related 7 (*ATG7*) or 5 (*ATG5*) gene exhibits phenotypes similar to those found in aging (Rubinsztein et al., 2011). Conversely, increase in autophagic activity has been associated with delayed aging in animal models (Rubinsztein et al., 2011; Tabibzadeh, 2023) as the restoring the expressions of autophagy genes can counteract age-related damage and decline (Yang et al., 2010; Bjedov et al., 2020; Hosoda et al., 2023; Huang et al., 2023). Unfortunately, the mechanisms by which autophagic components or processes decrease with age remain unclear (Chung and Chung, 2019), making it difficult to implement rejuvenation interventions impacting the altered mechanisms associated with autophagy.

Growing evidence supports that autophagy induced by calorie restriction (CR) has a substantial beneficial role (Chung and Chung, 2019); CR has been proven to increase both the health and lifespan in a wide range of animal models (Hwangbo et al., 2020) as well as support healthy human aging (Belsky et al., 2017). Therefore, CR is regarded as the gold standard for many aging intervention methods. Although CR has clearly diverse effects in counteracting the aging process, the exact mechanisms are still under investigation. Modulation of autophagy has a deep impact on cellular aging because it protects cellular functionality in several ways. In physiological conditions, autophagy plays crucial roles in inhibiting premature cell death by regulating the intracellular nutrient levels and metabolite availability, facilitating the turnover of cytoplasmic organelles, alleviating cellular stress, mitigating inflammation, preserving the self-renewal potential of stem cells, and maintaining the differentiation capacity and plasticity (Guo et al., 2022; Tabibzadeh, 2023) to modulate senescence (Tabibzadeh, 2023). This protection offered by autophagy is progressively lost during aging (Tabibzadeh, 2023).

### 4.2 Aging of the cardiovascular system

In the cardiocirculatory system, vascular aging is a complex process that causes structural and functional changes in the blood vessels and can be considered vascular remodeling. Vascular aging is characterized by increased arterial wall thickness and decreased lumen diameter, with an overall increase in the ratio of wall thickness to lumen diameter, reduced elasticity (increased stiffness), increased collagen, and reduced elastin deposition in the extracellular matrix (ECM) (Rizzoni et al., 2019; Koutouroushis and Sarkar, 2021). Four types of cells are involved in vascular remodeling: fibroblasts in the adventitial layer, vascular smooth muscle cells (VSMCs) in the median layer, endothelial cells (ECs) in the intimal layer, and macrophages in the blood stream (Qi et al., 2019). One of the main reasons for vascular remodeling is the transition of VSMCs from the contractile and low-proliferative phenotype to highly proliferative synthetic cells characterized by high production of ECM (Qi et al., 2019; Lehners et al., 2018). Aged vasculature often presents dysfunctional ECs with diminished production of nitric oxide, which is responsible for vascular dilatation, tone regulation, and inflammation inhibition. Furthermore, the senescence of ECs can result in reduced proliferation, which in turn allows VSMC migration and ECM deposition (Qi et al., 2019; Liu et al., 2024a).

The heart is also affected by structural and functional alterations with age and age-related changes, including reduced myocardial contractile capacity, wall thickening consequent to increased cardiomyocyte size, fibrosis (pathological deposition of ECM) leading to ventricular stiffness, stenosis (inflammation and calcification of the valves) resulting in narrowing and stiffness, and impaired conduction transmission with loss of the pacemaker cells (Obas and Vasan, 2018; Tracy et al., 2020; Koutouroushis and Sarkar, 2021). All these changes mediate the decline of cardiac functions and increase heart vulnerability to stress in the elderly. As a result, the risks of CVDs such as strokes, MI, atrial fibrillation, and atherosclerosis also increase (Koutouroushis and Sarkar, 2021; Chang et al., 2020; Yan et al., 2021). However, studies on cardiovascular remodeling and functioning during the process of aging (Zhang et al., 2023a; Zhao et al., 2024) are less common than studies about CVDs (Watanabe et al., 2020; Stassen et al., 2022; Elghazaly et al., 2023; Serio et al., 2023; Sheng et al., 2024; Kinoshita et al., 2024) because normal healthy aging is rarely studied as a disease-like entity (Jusic et al., 2022).

### 4.3 Role of autophagy in CVDs

Autophagy can both protect cardiovascular function and promote vascular remodeling. Protective autophagy often serves to maintain cardiovascular functions under physiological conditions, but excessive or dysregulated autophagy can contribute to disease development under pathological conditions (Mei et al., 2014; Sciarretta et al., 2018; Ren et al., 2023a). Therefore, modulating autophagy can be a successful strategy for counteracting age-induced vascular and cardiac remodeling to relieve CVDs. Herein, we describe recent studies (from the last 5 years) about the beneficial and detrimental roles of autophagy in the aging of the cardiovascular system and CVDs.

#### 4.3.1 Role of autophagy in vascular tissues

Several studies support the notion that autophagy critically regulates the proliferation, migration, and matrix secretion of the VSMCs (Qi et al., 2019; Koutouroushis and Sarkar, 2021) as well as inflammation and senescence of the ECs (Koutouroushis and Sarkar, 2021; Liu et al., 2024c). Autophagy has a beneficial role of delaying senescence in the vascular ECs. Conversely, autophagy plays a detrimental role of promoting phenotype switching in VSMCs, with a few exceptions (Fang et al., 2023; Shu et al., 2024; 2025; Jiang et al., 2025). Therefore, it is important to consider the cell type before determining if autophagy is detrimental or beneficial. Many studies highlight the importance of signaling pathway modulation to activate or inhibit autophagy. For example, downmodulation of PI3K/AKT/mTOR signaling is well known to promote autophagy (this pathway is also associated with longevity, aging, and cardiovascular health) (Koutouroushis and Sarkar, 2021). Next, we present recent results on autophagy modulation through regulation of the signaling pathways. In fact, investigations on the impact of autophagy via direct modulation of the autophagy-related proteins or studies on the impacts of autophagy on other cellular processes/pathways are scarce.

Li.et al. (2021a) associated the induction of autophagy with VSMC proliferation during hypertension. The mechanoresponsive nuclear envelope proteins are already associated with vascular remodeling in response to hypertension (Qi et al., 2016). Li et al. (2021b) demonstrated that suppression of lamina A/C and emerin can induce autophagy via the mTOR pathway, which in turn promotes VSMC proliferation and vascular remodeling. More recently, Shen et al. (2024a) studied angiotensin-II (Ang-II)-induced aortic dissection, which can be prevented by S-adenosylmethionine (SAM) through inhibition of autophagy and the cellular phenotypic switching of VSMCs; here, the activation of the PI3K/AKT/mTOR signaling pathway is the proposed molecular mechanism. Moreover, changes in transcription regulation were demonstrated to be involved in the autophagic modulation of vascular cells. During the development of aortic dissection (separation of the layers of the aortic wall), CCAAT/enhancer binding protein (C/EBP*α*) binding to the PIK3C2A promoter (gene encoding type II PI3Ks) can activate autophagy and phenotypic switching of the VSMCs from contractile to synthetic cells (Lu et al., 2022). The role of the C/EBP*α* transcription factor is still under investigation in VMSCs and has been recently reported to be associated with the regulation of vascular calcification (Chen et al., 2022a). Continuing with the transcription factors involved in the modulation of autophagy, the upregulation of NK2 homeobox 3 (NKX2-3), which is involved in tissue differentiation and organ development, has been shown to enhance autophagy through the AMPK/mTOR signaling pathway as well as modulate the proliferation and migration of VMSCs along with vascular remodeling (Zheng et al., 2021b). The forkhead box protein O (FOXO) transcription factors have multiple roles in the regulation of autophagy, as noted by Cheng (2019). FOXO3a promotes VSMC phenotype switching by enhancing autophagy in Ang-II-induced aortic aneurysms (Lu et al., 2021), while the peroxisome-proliferation-activated receptor γ (PPARγ) attenuates H_2_O_2_-induced senescence in VSMCs via the mTORC2/FOXO3a/autophagy signaling pathway (Kim et al., 2023). Furthermore, ECs have been considered in the evaluations of vascular senescence. Zhang et al. (2023a) investigated the roles of CD44, a cell surface adhesion molecule involved in angiogenesis and cardiac remodeling, in the senescence of vascular ECs. During aging, the upregulation of CD44 leads to reduced levels of PIK3R4 and PIK3C3, which are key components of the PI3K complex; this reduction in the activity of the PI3K complex results in a decline in autophagy and subsequent senescence of the ECs (Zhang et al., 2023a).

Aside from the PI3K/AKT/mTOR pathway that triggers autophagy, upregulation of heat shock protein-110 (HSP110) promotes proliferation, migration, and autophagy of pulmonary artery smooth muscle cells (PASMCs) during pulmonary hypertension. Here, HSP110 regulates the YAP/TAZ-TEAD4 pathway involved in cellular proliferation and angiogenesis. The TEA domain transcription factor 4 (TEAD4) regulates HSP110 transcription by binding to the HSP110 promoter (Liu et al., 2022). In arteriosclerosis obliterans (peripheral arterial presentation of atherosclerosis), downregulation of the Grb2-associated binder 1 (GAB1) protein has been shown to significantly increase autophagy in ECs through activation of the MAPK pathways, which in turn inhibit cell proliferation and migration (Qian et al., 2020). Interestingly, Yu et al. (2023a) recently discovered a new function of myosin 1b (MYO1B) in ECs; they showed that the expression of MYO1B increases during aging and is responsible for intracellular calcium homeostasis through its interaction with leucine-rich repeat kinase 2 (LRRK2), inducing the augmentation of intracellular calcium to impair autophagy, promote the senescence of ECs, and promote vascular aging (Yu et al., 2023a).

Although rarer than the analyses of signaling pathways and transcription factors regulating autophagy, we present some studies focusing on the direct modulation of autophagy through the regulation of autophagy proteins during aging or CVD. In Ang-II induced vascular remodeling, the overexpression of transmembrane member 16a (TMEM16a) ameliorates vascular remodeling and inhibits the proliferation of VSMCs. TMEM16a is a subunit of the calcium-activated chloride channels that can inhibit autophagy via modulation of the interactions between p62, BCL2, BECLIN1, and VPS34 while decreasing VSP34 activity (Lv et al., 2020). After coronary intervention, overexpression of methyltransferase-like 3 (METTL3) can increase autophagy by promoting the expressions of ATG5 and ATG7 proteins in VSMCs and thereby inhibiting vascular remodeling (Fang et al., 2023). Additionally, various enzymes may be involved in autophagy, such as nattokinase that possesses antioxidative and anti-inflammatory effects while suppressing the inflammation of ECs by inducing autophagy through activation of the transcription factor serum response factor (SRF) and glycoprotein thrombospondin 1 (THBS1), both of which are associated with inflammation regulation (Chiu et al., 2024).

Autophagy can also impact molecular and cellular mechanisms by degrading proteins or protein complexes to affect vascular remodeling. Yu et al. (2022) studied vascular remodeling after aortic allograft and demonstrated that autophagy activation upregulates the expression of the transcription factor sex-determining region Y box (SOX9) by degrading p27; p27 is a transcriptional corepressor that blocks the expression of SOX9 in association with p130 and E2F4; in turn, SOX9 promotes VMSCs of the synthetic phenotype (Yu et al., 2022). In conclusion, most recent studies have focused on the roles of autophagy in vascular remodeling in the context of specific pathologies. Only a few studies have investigated the contributions of autophagy to vascular aging (Yu et al., 2023a; Zhang et al., 2023b), although it is widely acknowledged that advanced age is one of the primary risk factors of CVDs.

#### 4.3.2 Role of autophagy in cardiac tissues

As in other tissues, autophagy in the heart decreases with aging. Since autophagy is required for the maintenance of cardiac structure and functions, it has been proposed that a decline in autophagy may be associated with the aging process of the heart (Obas and Vasan, 2018; Yan et al., 2021). Over the last 5 years, several studies have investigated the action mechanisms of molecules modulating CVDs (Guo et al., 2021; Aziz et al., 2022; Liao et al., 2022; Nagarajan et al., 2023; Shen et al., 2024b; Ou et al., 2024), and only a few of these works propose new pathways and mechanisms by which autophagy could be involved in cardiac aging and diseases. In the present review, we focus on these recent studies. Liu et al. (2024a) showed that decreased nuclear cardiac troponin I (cTnI) impairs autophagy during aging by downregulating the transcription factor Fos proto-oncogene, which in turn reduces ATG5 expression. Different studies have demonstrated that activation of autophagy is a prosurvival mechanism to reduce cellular stress and remove the organelles damaged during MI; therefore, enhancing autophagy is a promising mode of treatment for heart diseases (Sciarretta et al., 2018; Huang et al., 2023). For example, knockout mice for NOD-, LRR-, and pyrin-domain-containing protein 3 (NLRP3) showed inhibition of the PI3K/AKT/mTOR pathway and enhanced autophagy, resulting in reduced cardiac damage (Marín-Aguilar et al., 2020). Alternatively, the activation of glycogen synthase kinase 3 beta (GSK-3β) promoted autophagy through the phosphorylation of Unc-51 like autophagy activating kinase 1 (ULK1) to prevent cardiac aging (Chen et al., 2021a).

### 4.4 Roles of ncRNAs in autophagy in vascular and cardiac cancers

Vascular and cardiac tissues are very susceptible to alterations that can lead to the development of tumors, and autophagy plays a dual role in tumors. During the early phases of tumor formation, autophagy functions as a tumor suppressor to restore homeostasis and eliminate cellular aberrations. Indeed, misfolded proteins and organelles as well as reactive oxygen species (ROS) are removed through basal autophagy, thus avoiding genomic damage that could lead to carcinogenesis. Conversely, in the later phases, autophagy may either support or facilitate tumor growth by allowing the tumor cells to adapt to a stressful environment. Vascular tumors, such as hemangiomas and hemangioendotheliomas, are formed during infancy and childhood but are usually left as is until complete involution. For a comprehensive review on the ncRNAs involved in infantile hemangiomas, see Wang et al. (2024a). Although the ncRNAs directly involved in autophagy have not been identified yet, it is interesting to note that several ncRNAs involved in infantile hemangiomas are involved in apoptosis regulation (Wang et al., 2024a). This represents a response to autophagy since excessive autophagy in the context of specific diseases could result in cell death through apoptosis (Xi et al., 2022). Unlike hemangiomas and hemangioendotheliomas, angiosarcomas can arise at any age and predominantly affect elderly persons, with poor long-term prognosis (Sepulveda and Buchanan, 2014; Ota et al., 2023; Wagner et al., 2024a). The incidence of angiosarcoma remains uncertain, ranking between 0.15 and 0.33 per 100,000 person-years (Wagner et al., 2024b). The significance of miRNAs in angiosarcoma is demonstrated by the evidence that mutations in the RNA helicase/RNase III Dicer are associated with angiosarcoma pathogenesis (Loh et al., 2023). Dicer is a crucial protein involved in the production of miRNAs; although the influences of miRNAs in angiosarcomas have been discussed recently (Modarresi Chahardehi et al., 2024), there are no established associations between ncRNAs and autophagy in angiosarcomas. Notably, the contribution of autophagy to angiosarcomas remains poorly understood. Both inhibition and a high level of autophagy have the potential to prevent tumorigenesis (Suzuki et al., 2022; Yang et al., 2023). An intriguing observation in sarcomas is that their treatment, including radiotherapy, chemotherapy, immunotherapy, and targeted therapy, induces modulation of both miRNA and lncRNA expressions; these modulations are guided by tumor resistance (Chen et al., 2022b). Consequently, ncRNAs have central roles in not only treatment but also monitoring of the treatment effects. Primary heart cancer is one of the rarest neoplastic entities, with an incidence of 0.33%. The low incidence of heart cancer may be related to the mechanical forces exerted by the contraction of cardiomyocytes that could disrupt the adhesion and survival of cancer cells, including cells that may have adhered to the intramuscular endothelium (Kump, 2024). Aside from their rarity, the prognosis of cardiac tumors is usually poor with an overall survival of 12–17 months after diagnosis. The diagnosis is usually made late in such cases because the symptoms often begin after a stroke or an ischemic attack caused by detached tumor tissue or thrombus (Geronikolou et al., 2021). For example, myxoma is the most common type of cardiac cancer that could embolize and consequentially be lethal (Campisi et al., 2022). Cardiac tumors can develop in adults between the ages of 50 and 70 years, as exemplified by myxoma and malignant mesothelioma. Alternatively, they can manifest in infancy and childhood, as in the case of rhabdomyoma, rhabdomyosarcoma, and fibroma (Campisi et al., 2022). As discussed previously, during the late phases of cancer, aberrant autophagy increases intracellular stress and leads to DNA damage, which in turn could promote cancer progression. The role of autophagy in adult cardiac cancer is not clear since the related studies are scarce. It has been reported that myxoma can upregulate autophagy (Jantuan et al., 2021; Sramek et al., 2021) and that there is close interplay between autophagy and the immune system in this cancer (Sramek et al., 2021). Inhibition of autophagy in myxoma has not yet been investigated; nevertheless, autophagy is a key resistance mechanism in rhabdomyosarcoma. Indeed, autophagy inhibitors can be beneficial in combination with cancer therapy (Zarrabi et al., 2023).

Cardiac myxoma is a significant contributor to stroke in young adults, and its diagnosis poses challenges in patients presenting with stroke owing to the absence of diagnostic biomarkers. To identify the ncRNAs involved in ischemic stroke caused by myxoma, Ma et al. (2025) compared tumor tissues between patients with cardiac myxoma-related ischemic stroke (CM-IS) and patients with cardiac myxoma (CM). Furthermore, since they were interested in tumor communication, these authors evaluated miRNA, lncRNA, and mRNA in exosomes purified from the plasma samples of patients with CM-IS and CM. In the plasma samples, they identified 74 differentially expressed miRNAs, 12 lncRNAs, and 693 mRNAs, while in the tumor-derived tissue samples, they identified 61 miRNAs, 67 lncRNAs, and 433 mRNAs (Ma et al., 2025). Notably, among the upregulated miRNAs in the CM-IS-derived plasma, miR-486 and miR-96 were also upregulated in the CM-IS tissue samples, supporting the idea that CM-IS tissues are responsible for their secretion; miR-96 promotes MI-induced apoptosis by targeting antiapoptotic genes (Wang et al., 2021), while miR-486 exhibits protective roles against cardiac ischemia-reperfusion (I/R) injury and myocardial apoptosis (Bei et al., 2022). However, the results concerning the secreted miRNAs and their potential roles in regulating apoptotic processes remain inconclusive. The identified miRNAs secreted by the CM-IS tissues may be involved in both the induction and protection of apoptosis; this discrepancy may be influenced by the absence of gender differences in the study and primarily by the heterogeneity of the patient ages. Most CM-IS patients enrolled in the study were approximately 55 years of age, with the extremes being 38-year-old and 70-year-old subjects. Notably, the older patient had the smallest tumor size. Interestingly, miR-486 has been reported to modulate cardiomyocyte cell size and inflammatory responses in heart failure (Verjans et al., 2019). The pleiotropic activity of miRNA may be another factor influencing these variable results. Hence, understanding the types of cells that express the studied miRNAs could provide insights into their functions in relation to the genes expressed by the specific cell types. Indeed, different cells expressing different genes could be associated with the functions of miRNAs. Lastly, one aspect that is not considered in the above studies is the subcellular localization of miRNAs and their compartmentalisation, which could affect their availability and consequently their activities.

### 4.5 Non-coding RNAs in aging and age-related diseases

The Encyclopedia of DNA Elements project has unveiled that less than 3% of the human genome codes for proteins (ENCODE Project Consortium, 2012) but it is pervasively transcribed into RNA molecules known as ncRNAs (Jensen et al., 2013). Among these, miRNAs, lncRNAs, and circular RNAs (circRNAs) have been shown to be involved in the regulation of gene expression to control cellular processes (Panni et al., 2020). Herein, we review the most recent studies (from the last 5 years) that highlight the involvement of ncRNAs in the modulation of autophagy with the aim of identifying those that can serve as novel therapeutic targets for CVDs.

### 4.6 MicroRNAs modulating autophagy in CVDs

The miRNAs are often short ncRNAs of approximately 19–22 nucleotides length that regulate gene expressions by repressing translation or inducing mRNA degradation of the target transcripts. This regulation is typically achieved through sequence-specific binding to the 3′ untranslated region (3′UTR) of the target mRNA (Bushati and Cohen, 2007). Studies have recognized miRNAs as important regulators of aging processes (Kinser and Pincus, 2020) and pathogenesis of CVDs (Guo et al., 2022). The following studies underscore the significance of miRNAs in both direct and indirect regulation of autophagy. Notably, miRNAs exert an indirect influence on autophagy by targeting mRNAs encoding proteins involved in the signaling pathways governing autophagy (i.e. mTOR pathway).

#### 4.6.1 MicroRNAs modulating autophagy in vascular tissues

**FIGURE 3:**
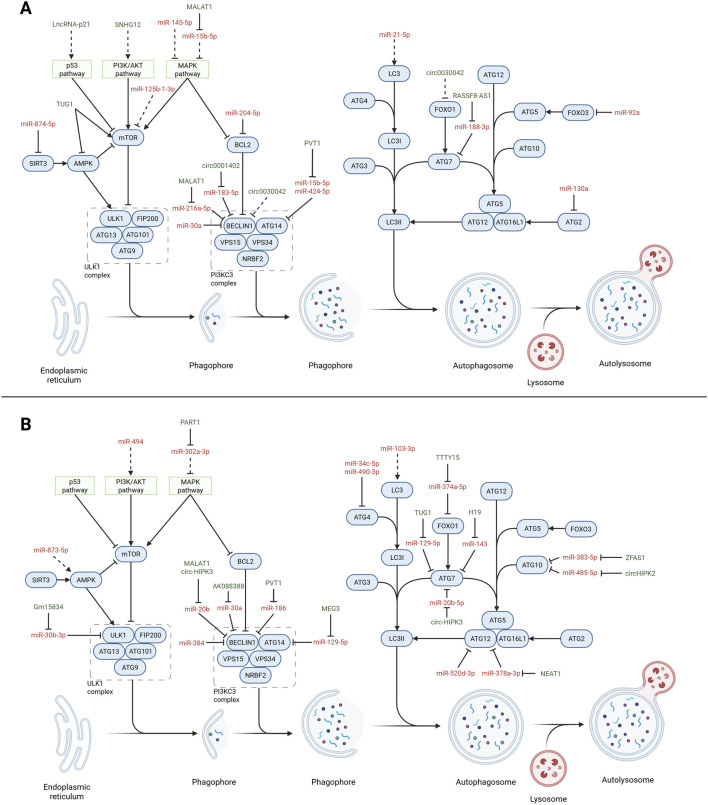
Non-coding RNAs (ncRNAs) affecting autophagy in CVDs. Overview of the autophagy pathway (blue) highlighting the interactions with microRNAs (red) and long non-coding RNAs (lncRNAs; green) in regulating the autophagy process in CVDs. The dotted lines indicate indirect interactions. **(A)** Non-coding RNAs involved with vascular tissues. MicroRNAs: miR-874-5p (Zhang et al., 2020b), miR-204-5p (Tian et al., 2024), miR-183-5p (Lin et al., 2024), miR-30a (Shi et al., 2022), miR-15b-5p (Zhang et al., 2023a), miR-424-5p (Zhang et al., 2023b), miR-188-3p (Song et al., 2023), miR-92a (Cao et al., 2024), miR-130a (Zheng et al., 2021a), miR-125b-1-3p (Chen et al., 2024a), miR-145-5p (Zhang et al., 2022a), miR-21-5p (Ke et al., 2022), and miR-216a-5p (Wang et al., 2019b). Long non-coding and circular RNAs: SNHG12 (Li et al., 2019b), MALAT1 (Wang et al., 2019c; Zhu et al., 2019), PVT1 (Zhang et al., 2023c), RASSF8-AS1 (Song et al., 2023), circ0001402 (Lin et al., 2024), and circ00300442 (Yu et al., 2021). **(B)** Non-coding RNAs involved with cardiac tissues. MicroRNAs: miR-30a (Wang et al., 2019a), miR-873-5p (Zhu et al., 2024), miR-129-5p (Mi et al., 2023), miR-34c-5p (Zhang et al., 2022b), miR-374a-5p (Chen et al., 2021c), miR-520d-3p (Wu et al., 2021c), miR-383-5p (Liu et al., 2024a), miR-485-5p (Zhou et al., 2020), miR-103-3p (Xue et al., 2023), miR-384-5p (Zhang et al., 2019), miR-490-3p (Wu et al., 2021b), miR-494 (Ning et al., 2020), miR-143 (Lv et al., 2021), miR-20b (Wang et al., 2019c; Qiu et al., 2021), miR-378a-3p (Zhao et al., 2020), miR-302a-3p (Zeng et al., 2024), and miR-186 (Ouyang et al., 2020). Long non-coding and circular RNAs: AK088388 (Wang et al., 2019a), TUG1 (You et al., 2020; Tan et al., 2023), H19 (Lv et al., 2021), MALAT1 (Wang et al., 2019c), NEAT1 (Zhao et al., 2020), PART1 (Zeng et al., 2024), MEG3 (Mi et al., 2023), TTTY15 (Chen et al., 2021c), ZFAS1 (Liu et al., 2024b), circHIPK2 (Zhou et al., 2020), and circ-HIPK3 (Qiu et al., 2021).Created with Biorender.com.

Aberrant autophagy can promote vascular remodeling and development of CVDs. The expression of myocardin is essential for maintaining the contractile phenotype of VSMCs as it inhibits autophagy by the miR30a/BECLIN1 axis (Shi et al., 2022). Similarly, miR-130a inhibits autophagy by targeting ATG2B in the VSMCs (Zheng et al., 2021b). MiR-125b-1-3p ameliorates atherosclerosis in mice by enhancing autophagy in the VMSCs via the RRAGD/mTOR/ULK1 axis. Ras-related GTP binding D (RRAGD) is a member of the Rag GTPase family that mediates mTOR signaling in autophagy regulation (Chen et al., 2024a). Moreover, miR-145-5p promotes autophagy via the AMPK/mTOR/ULK1 pathway by targeting calcium-/calmodulin-dependent protein kinase II delta (CaMKIIδ) in atherosclerotic VSMCs; CaMKIIδ is a serine/threonine protein kinase that can activate AMPK (Zhang et al., 2022a). MiR-874-5p targets SIRT3 to induce autophagy in the PASMCs under hypoxic pulmonary hypertension; SIRT3 has recently been reported to be involved in the regulation of autophagy (Zhang et al., 2020b). However, miR-92a expression is associated with CVDs and inhibits autophagy by targeting FOXO3 in the ECs (Cao et al., 2024). Furthermore, miR-483-5p targeting TIMP metallopeptidase inhibitor 2 (TIMP2) promotes atherosclerosis development and endothelial dysfunction by inhibiting autophagy. In fact, TIMP2 downregulation is associated with progression of CVDs (Zhu et al., 2023). For comprehensive reviews on the ncRNAs involved atherosclerosis, the readers are referred to other works (Yuan et al., 2021; Singh et al., 2022). Figure 3A presents a summary of the miRNAs influencing autophagy in CVDs, while Table 6 provides a comprehensive overview of the studies reported herein, including the pathological conditions and models utilized.

**TABLE 6:** MicroRNAs regulating autophagy in cardiovascular diseases (CVDs).

MicroRNAs	Condition/Disease	Models	Non-coding RNA roles	References
miR-103-3p	HF	HL-1 CMs treated with Ang-II	Promotes autophagy by targeting HLF transcription factor and FYCO1 that interact with LC3	Xue et al. (2023)
miR-125b-1-3p	AS	Apoe ^−/−^ mouse and MOVAS cells (mouse VMSCs)	Promotes autophagy by targeting RRAGD, which is a mediator of the mTOR signaling pathway	Chen et al. (2024a)
miR-130	AS	Human VSMCs	Inhibits autophagy by targeting ATG2B	Zheng et al. (2021b)
miR-145-5p	AS	Human aortic VSMCs	Promotes autophagy via the AMPK/mTOR/ULK1 pathway by targeting CaMKIIδ	Zhang et al. (2022b)
miR-204-5p	AS	ApoE ^−/−^ mouse, human umbilical vein ECs, and human aortic SMCs	Targets BCL2 in VSMCs	Tian et al. (2024)
			Targets RUNX2 in ECs	
miR-21-5p	AS	Endothelial colony-forming cells	Promotes autophagy by targeting SIPA1L2 that interacts with LC3	Ke et al. (2022)
miR-30a	Vascular proliferative diseases	Human aortic VSMCs	Inhibits autophagy by targeting BECLIN1	Shi et al. (2022)
miR-34c-5p	Cardiac hypertrophy	Isoprenaline-treated mice and rat CMs	Inhibits autophagy by targeting ATG4B	Zhang et al. (2022a)
miR-384-5p	I/R-induced myocardial injury	H/R treated H9C2 CMs	Inhibits autophagy by targeting BECLIN1	Zhang et al. (2019)
miR-483-5p	AS	Human umbilical vein ECs treated with ox-LDL	Inhibits autophagy by targeting TIMP2	Zhu et al. (2023)
miR-490-3p	I/R-induced myocardial injury	I/R mouse model	Inhibits autophagy by targeting ATG4	Wu et al. (2021c)
miR-494	I/R-induced myocardial injury	H/R treated H9C2 CMs	Inhibits autophagy by targeting SIRT1	Ning et al. (2020)
miR-520d-3p	H/R-induced myocardial injury	I/R injury rat model and H/R-treated human CMs	Inhibits autophagy by targeting ATG12	Wu et al. (2021b)
miR-873-5p	Myocardial infarction	MSCs	Promotes autophagy by regulating the AMPK signaling pathway	Zhu et al. (2024)
miR-874-5p	Hypoxic pulmonary hypertension	Rats exposed to chronic hypoxia and PASMCs	Promotes autophagy by targeting SIRT3	Zhang et al. (2020b)
miR-92a	CVDs	EA.hy926 cells (ECs)	Inhibits autophagy by targeting FOXO3	Cao et al. (2024)

ECs: endothelial cells, CMs: cardiomyocytes, MSCs: mesenchymal stem cells, SMCs: smooth muscle cells, VSMCs: vascular smooth muscle cells, PASMCs: pulmonary artery smooth muscle cells, ox-LDL: oxidized low-density lipoprotein, Ang-II: angiotensin-II, H/R: hypoxia/reoxygenation, I/R: ischemia/reperfusion, AS: atherosclerosis, HF: heart failure.

#### 4.6.2 MicroRNAs modulating autophagy in cardiac tissues

The roles of miRNAs in I/R-induced myocardial injuries have been widely investigated; miR-494 (Ning et al., 2020), miR-384-5p (Zhang et al., 2019), and miR-490-3p (Wu et al., 2021a) have been shown to alleviate myocardial injury via autophagy inhibition by targeting sirtuin 1 (SIRT1), BECLIN1, and autophagy related 4A cysteine peptidase (ATG4), respectively. Moreover, autophagy inhibition promotes hypertrophy via the miR-34c-5p/ATG4B axis (Zhang et al., 2022b). The overexpression of miRNA-520d-3p inhibits the expression of autophagy related 12 (ATG12) to attenuate apoptosis in myocardial cells after hypoxia/reoxygenation (H/R) (Wu et al., 2021a). In the cell model for heart failure progression, miR-103-3p has been shown to promote autophagy by targeting the hepatic leukemia factor (HLF) transcription factor as well as FYVE and coiled-coil domain autophagy adaptor 1 (FYCO1) that directly interact with LC3 to increase autophagic flux (Xue et al., 2023). Finally, mesenchymal stem cells (MSCs) may have cardioprotective properties through the promotion of angiogenesis following infarction. MiR-873-5p suppression in the MSCs enhances autophagy by regulating the AMPK signaling pathway (Zhu et al., 2024). The miRNAs regulating autophagy in CVDs are shown in Figure 3B and Table 6, including the pathological conditions and models used.

#### 4.6.3 Circulating ncRNAs

The potential roles of circulating miRNAs in cell–tissue communication are strongly supported by their stability related to their abilities to associate with lipoproteins and proteins or to remain within vesicles that allow miRNAs to be exported or imported from the cells through mechanisms involving vesicle and protein vector trafficking (Mori et al., 2019). Extracellular vesicles secrete not only miRNAs but also mRNAs, lncRNAs, and circRNAs (Kim et al., 2017). Endothelial colony-forming cells are ECs that mediate vascular repair and secrete exosomes containing miR-21-5p in atherosclerosis; these exosomes deliver miR-21-5p to target signal induced proliferation associated 1 like 2 (SIPA1L2) that interacts with LC3 and rescues autophagy in the ECs (Ke et al., 2022). Recently, Tian et al. (2024) investigated the communication between VSMCs and ECs. During atherosclerosis, activation of autophagy in the ECs induces packing and secretion of miR-204-5p; this targets BCL2 when absorbed by the ECs and runt-related transcription factor 2 (RUNX2) when absorbed by the VSMCs to alleviate smooth muscle cell calcification (Tian et al., 2024). In fact, RUNX2 has been reported to have a detrimental role in the cardiovascular system (Chen et al., 2021b). Notably, several lncRNAs were identified in exosomes released from MSCs treated with atorvastatin (Huang et al., 2020); these findings hold significant importance because atorvastatin is commonly prescribed to prevent CVD in individuals with abnormal lipid levels. Furthermore, exosomes are crucial for mediating cardioprotective mechanisms (Wei et al., 2021). Consequently, statins not only modulate HMG-CoA reductase (HMGCR) but also influence the secretion of lncRNAs that could synergistically contribute to physiological processes. This discovery presents compelling evidence of a positive therapeutic response to a pharmacological intervention that is not specifically targeted to lncRNAs but rather modulates their activity, thereby underscoring the physiological relevance. A summary of the studies reported herein is presented in Table 6.

### 4.7 Long ncRNAs modulating autophagy in CVDs

The lncRNAs are a heterogeneous class of regulatory ncRNAs that are poorly conserved among species; lncRNAs are subdivided into other categories according to their genomic context or function (Alessio et al., 2020). They regulate gene expressions at all levels of genome activity (transcriptional, RNA processing, translation, and post-translation) by interacting with DNA, RNA, or proteins (Mattick et al., 2023). LncRNAs are the basis of important pathological processes in aging and age-related diseases (He et al., 2018). To date, the proposed mechanisms primarily focus on lncRNAs functioning as competitive endogenous RNAs (ceRNAs) that compete with targets for miRNA binding; ceRNAs can contact miRNAs to modulate their availability within cells and ability to bind to the mRNA targets (Giza et al., 2014). Hence, ceRNAs are also called “sponges.” LncRNAs can modulate autophagy-related protein expressions or impact the signaling pathways to regulate autophagy by sponging miRNAs. The following sections highlight the importance of ncRNAs in the modulation of cellular processes and autophagy as well as provide a guidance for studies that on the use of the same RNAs as therapeutic agents. Indeed, unlike miRNAs that have multiple targets, lncRNAs offer more specificity at the expense of less conservation of their primary structures across species.

#### 4.7.1 Long ncRNAs modulating autophagy in vascular tissues

In the human aortic VSMC model of atherosclerosis, the lncRNA RASSF8 antisense RNA 1 (RASSF8-AS1) sponges miR-188-3p to elevate autophagy-related 7 (ATG7) expression and induce autophagy (Song et al., 2023). Similarly, the lncRNA plasmacytoma variant translocation 1 (PVT1) enhances autophagy to alleviate hypoxia-induced apoptosis of ECs by binding to miR-15b-5p and miR-424-5p, thereby avoiding the miRNAs targeting autophagy-related 14 (ATG14) (Zhang et al., 2023c). In several cases, dysfunction of ECs can be caused by inflammation; the potential of lncRNAs in modulating inflammation and subsequent autophagy has been demonstrated. LINC00346 acts as a miRNA-637 sponge to positively regulate the expression of NLRP1, a member of the NLR family (Ge et al., 2023) of proteins that are involved in the immune system to help regulate the process of inflammation. Recently, the serum level of NLRP1 was shown to be positively related to unstable angina (Zong et al., 2023). Contrarily, downregulation of the lncRNA metastasis-associated lung adenocarcinoma transcript 1 (MALAT1) impacts the level of miR-19b-3p, increasing its availability and permitting the inhibition of hypoxia-inducible factor 1α (HIF-1α) (Liu et al., 2020). HIF-1α is known to be involved in apoptosis, autophagy, and inflammation; therefore, the modulation of MALAT1 may be an alternative method of reducing apoptosis, autophagy, and inflammation. Wang et al. (2019a) demonstrated that MALAT1 has a direct impact on autophagy; in the HUVEC model of atherosclerosis, this lncRNA sponges miR-216a-5p to positively regulate BECLIN1 and promote autophagy (Wang et al., 2019b). Moreover, MALAT1 contributes to atherosclerosis by inhibiting autophagy in the endothelial progenitor cells via miR-15b-5p/MAPK1. Mitogen-activated protein kinase 1 (MAPK1), whose expression is associated with atherosclerosis, can activate the mTOR signaling pathway to inhibit autophagy (Zhu et al., 2019).

The previously reported results describe the function of lncRNAs as miRNA sponges, indicating their localization within the cytoplasm of cells. However, this location is not exclusive for lncRNAs as they may also be present in the nucleus, where they interact with the chromatin and transcription machinery to regulate gene expressions. For example, the overexpression of lncRNA-p21 enhances autophagy and attenuates senescence in Ang-II-induced damage to the ECs. The lncRNA-p21 activates the SESN2/AMPK/TSC2 pathway by promoting the transcriptional activity of p53 (Li et al., 2021a); p53 is an important modulator of p21 that regulates cell cycle and senescence. Both lncRNA-p21 and the lncRNA taurine upregulated gene 1 (TUG1) are modulated in Ang-II-induced damage to the ECs; TUG1 downregulation reduces vascular damage by permitting the action of miR-9-5p, which represses the concentration of the transcript for the C-X-C motif chemokine receptor 4 (CXCR4) (Shi et al., 2024). CXCR4 is a CXC chemokine receptor involved in different signalling transductions of ECs. Moreover, TUG1 knockdown in the EC model of atherosclerosis was reported to promote autophagy via the AMPK/mTOR pathway (You et al., 2020). Comprehensive reviews on the ncRNAs involved in atherosclerosis are available elsewhere (Yuan et al., 2021; Singh et al., 2022). Figure 3A summarizes the impacts of lncRNAs on the autophagy pathway in CVDs. Tables 7, 8 report the pathological conditions and models used for the descriptions of the lncRNA functions in vascular tissues and cardiomyocytes, respectively.

**TABLE 7:** Long non-coding RNAs regulating autophagy in endothelial and vascular muscle cells.

Long non-coding RNAs	Condition/Disease	Models	Non-coding RNA roles	References
AC136007.2	I/R injury	I/R injury rat model; oxygen-glucose deprivation and reoxygenation-treated SH-SY5Y cells	Inhibits autophagy via the PI3K/AKT/mTOR signaling pathway	Liu et al. (2021)
LINC00346	AS	Human umbilical vein ECs treated with ox-LDL	Regulates inflammation by sponging miR-637 to modulate NLRP1 expression	Ge et al. (2023)
LncRNA-p21	Hypertension	Human endothelial progenitor cells	Activates the SESN2/AMPK/TSC2 pathway by promoting transcriptional activity of p53	Li et al. (2021c)
MALAT 1	AS	Human umbilical vein ECs treated with ox-LDL	Promotes autophagy by sponging miR-216a-5p to induce BECLIN1	Wang et al. (2019b)
MALAT1	Hypoxia	Human umbilical vein ECs in hypoxic conditions	Regulates inflammation by sponging miR-19b-3p to modulate HIF-1α expression	Liu et al. (2020)
MALAT1	AS	Endothelial progenitor cells	Inhibits autophagy by sponging miR-15b-5p to induce MAPK1	Zhu et al. (2019)
RASSF8-AS1	AS	Human aortic VSMCs	Promotes autophagy by sponging miR-188-3p to induce ATG7	Song et al. (2023)
SNHG12	I/R injury	Rat model of middle cerebral artery occlusion, I/R treated MSCs, and rat brain microvascular ECs	Inhibits autophagy via the PI3K/AKT/mTOR signaling pathway	Li et al. (2019b)
TUG1	Hypertension	Ang-II-treated human umbilical vein ECs and spontaneously hypertensive rat	Sponges miR-9-5p to induce CXCR4 expression	Shi et al. (2024)
TUG1	AS	Human umbilical vein ECs	Promotes autophagy via the AMPK/mTOR pathway	You et al. (2020)

ECs: endothelial cells, MSCs: mesenchymal stem cells, SMCs: smooth muscle cells, VSMCs: vascular smooth muscle cells, ox-LDL: oxidized low-density lipoprotein, Ang-II: angiotensin-II, I/R: ischemia/reperfusion, AS: atherosclerosis.

**TABLE 8:** Long non-coding RNAs regulating autophagy in cardiomyocytes.

Long non-coding RNAs	Condition/Disease	Models	Non-coding RNA roles	References
AK088388	Myocardial I/R injury	H/R treated HL-1 CMs	Promotes autophagy by sponging miR-30a to induce BECLIN1	Wang et al. (2019a)
DCRF	Diabetic cardiomyopathy	Diabetic rat models and primary rat CMs	Promotes autophagy via the miR-551b-5p/PCDH17 axis	Feng et al. (2019)
FOXD3-AS1	Myocardial I/R injury	Oxygen-glucose deprivation and reoxygenation-treated H9C2 CMs	Promotes autophagy by activating the NFκB/COX2/iNOS signaling pathway	Tong et al. (2019)
H19	Myocardial I/R injury	I/R injury mouse model and H/R-treated HL-1 CMs	Promotes autophagy by sponging miR-143 to induce ATG7	Lv et al. (2021)
LncRNA 2810403D21Rik/Mirf	Myocardial infarction	Neonatal mice CMs and myocardial infarction mouse model	Inhibits autophagy by sponging miR-26a to induce USP15	Liang et al. (2020)
LncRNA Gm15834	Myocardial hypertrophy	Ang-II treated HL-1 and AC16 CMs; transverse aortic constriction mouse model	Promotes autophagy by sponging miR-30b-3p to induce ULK1	Song et al. (2021)
MALAT1	Myocardial I/R injury	Oxygen-glucose deprivation and reoxygenation-treated H9C2 CMs	Promotes autophagy by sponging miR-20b to induce BECLIN1	Wang et al. (2019c)
MEG3	HF	Isoprenaline-treated mouse and H9C2 CMs treated with H_2_O_2_	Promotes autophagy by sponging miR-129-5p to induce ATG14	Mi et al. (2023)
NEAT1	Myocardial I/R injury	Primary rat CMs in hypoxia condition	Promotes autophagy by sponging miR-378a-3p to induce ATG12	Zhao et al. (2020)
PART1	Myocardial I/R injury	H/R-treated AC16 CMs	Promotes autophagy by sponging miR-302a-3p to induce TFAP2C	Zeng et al. (2024)
PVT1	Myocardial I/R injury	H/R-treated AC1 CMs	Promotes autophagy by sponging miR-186 to induce BECLIN1	Ouyang et al. (2020)
TTTY15	Myocardial I/R injury	I/R injury mouse model and H/R-treated H9C2 CMs	Promotes autophagy by sponging miR-374a-5p to induce FOXO1	Chen et al. (2021c)
TUG1	HF	AC16 cells CMs treated with H_2_O_2_	Promotes autophagy by sponging miR-129-5p to induce ATG7	Tan et al. (2023)
XIST	Myocardial I/R injury	I/R injury mouse model and H/R-treated H9C2 CMs	Inhibits autophagy via the miR-133a/SOCS2 axis	Li et al. (2019b)
ZFAS1	Hypoxia-induced myocardial injury	H9C2 CMs in hypoxic conditions	Promotes autophagy by sponging miR-383-5p to induce ATG10	Liu et al. (2024c)

CMs: cardiomyocytes, H/R: hypoxia/reoxygenation, I/R: ischemia/reperfusion, HF: heart failure.

#### 4.7.2 Long ncRNAs modulating autophagy in cardiac tissues

Previously, we discussed the involvement of lncRNAs in the modulation of autophagy in endothelial and vascular cells; now, we extend this discussion to myocardial cells. The lncRNA maternally expressed 3 (MEG3) interacts with miR-129-5p, permitting upregulation of ATG14 in H_2_O_2_-treated cardiomyocytes (Mi et al., 2023). Therefore, MEG3 downregulation increases autophagy and reduces apoptosis. It has also been demonstrated that the transcription factor ETS proto-oncogene 2 (ETS2) promotes expression of TUG1 in cardiomyocytes under oxidative stress conditions (Tan et al., 2023); in this work, the stress condition was used as the cell model for heart failure to demonstrate that TUG1 interacts with miR-129-5p to increase the expression of ATG7. Here, the authors showed that TUG1 overexpression reverses ETS2 knockdown-mediated inhibition of cardiomyocyte apoptosis and autophagy. Indeed, ETS2 is a transcription factor involved in development and apoptosis; therefore, they suggested that the modulation of ETS2 expression may be a new therapeutic approach for the treatment of heart failure. One of the common risks factors for CVDs is diabetes, and diabetic cardiomyopathy (DCM) is a major complication of this metabolic disease. The lncRNA DCM-related factor (DCRF) is associated with the development of DCM and regulates autophagy by acting as the ceRNA of miR-551b-5p; here, DCFR binds to miR-551b-5p to upregulate protocadherin-17 (PCDH17) that increases autophagy and has been reported to aggravate heart diseases (Feng et al., 2019). Song et al. (2021) described the direct impact of the lncRNA Gm15834 on autophagy; in the cardiac hypertrophic mouse model, knockdown of the lncRNA Gm15834 was found to inhibit autophagy by downregulating ULK1 through the release of miR-133a from Gm15834.

During ischemic attacks or strokes, the heart undergoes H/R stress that can induce myocardial injury; in this condition, autophagy has a detrimental role and induces the death of cardiomyocytes (Ma et al., 2015). During myocardial injury induced by hypoxia, the lncRNA ZNFX1 antisense RNA 1 (ZFAS1) promotes autophagy to positively regulate ATG10 by sponging miR-383-5p (Liu et al., 2024c); here, the authors proposed the possibility of using ZFAS1 as a therapeutic target for myocardial injury. The H/R cardiomyocyte model was also used by Ouyang et al. (2020) to investigate the role of the lncRNA PVT1 after myocardial I/R injury, where PVT1 downregulation was shown to alleviate H/R injury by inhibiting autophagy via the miR-186/BECLIN1 axis; the expression of BECLIN1 can be modulated by MALAT1/miR-20b in cardiomyocytes (Wang et al., 2019c). In addition, H19 (Lv et al., 2021) and nuclear enriched abundant transcript 1 (NEAT1) (Zhao et al., 2020) were shown to promote autophagy in I/R-induced myocardial injury models, upregulating ATG7 by targeting miR-143 and ATG12 by targeting miR-378a-3p. Conversely, the lncRNA X-inactive specific transcript (XIST) inhibits autophagy via the miR-133a/SOCS2 axis; however, the interactions between autophagy and the suppressor of cytokine signaling 2 (SOCS2) need to be elucidated (Li et al., 2019a). Liang et al. (2020) described the involvement of the lncRNA 2810403D21Rik/AK007586/Mirf (myocardial infarction regulatory factor) in macroautophagy/autophagy inhibition through modulation of miR-26a that targets the ubiquitin-specific peptidase 15 (USP15) transcript to alleviate ischemic-stress-induced cardiac injury. Therefore, the lncRNA Mirf could be a therapeutic target for counteracting the detrimental effects on autophagy after its transcriptional activation.

Similar to miRNAs, lncRNAs impact different signaling pathways to regulate autophagy in cardiac diseases. The lncRNA testis expressed transcript Y-linked 15 (TTTY15) acts as a ceRNA for miR-374a-5p to negatively regulate forkhead box O1 (FOXO1); FOXO1 is necessary for autophagy induction to alleviate myocardial injury, so the induction of TTTY15 during myocardial I/R injury is detrimental for cardiac recovery (Chen et al., 2021c). Here, another lncRNA is overexpressed in myocardial I/R injury, namely, prostate androgen regulated transcript 1 (PART1), whose function was described by Zeng et al. (2024). These authors showed that PART1 interacts with miR-302a-3p to allow upregulation of the transcription factor activating enhancer binding protein 2C (TFAP2C), which is a member of the AP2 family of transcription factors. TFAP2C regulates dual specificity phosphatase 5 (DUSP5), which is the phosphatase of ERK1/2 that suppresses I/R-stimulated autophagy and apoptosis (Zeng et al., 2024). Both studies provide evidence regarding the importance of lncRNAs for alleviating cardiomyocyte apoptosis and autophagy induced by I/R injury (Chen et al., 2021c; Zeng et al., 2024). Similarly, the lncRNA AK088388 regulates autophagy through miR-30a (Wang et al., 2019a); here, the authors showed that miR-30a expression is downregulated, while the expressions of AK088388, BECLIN1, and LC3-II are upregulated in the H/R cardiomyocytes; moreover, miR-30a targets both AK088388 and BECLIN1. The authors demonstrated that targeting AK088388 using siRNAs and upregulating miR-30a expression using miRNA mimics can enhance the viability of the H/R cardiomyocytes. These findings support the application of ncRNAs for potentially modulating cardiomyocyte viability through autophagy regulation. The lncRNA FOXD3 antisense RNA 1 (FOXD3-AS1) promotes autophagy during myocardial I/R injury (Tong et al., 2019); its upregulation enhances the expressions of LC3 II, BECLIN1, and ATG5 along with downregulation of p62 expression in H9C2 (embryonic rat heart tissue) cells. Although this finding was only based on *in vitro* experiments, it supports the idea that autophagy may also be modulated by downregulation of FOXD3-AS1. For a comprehensive review on the lncRNAs involved cardiac hypertrophy and heart failure, we refer readers to Mably and Wang (2024). Figure 3B summarizes the direct impacts of lncRNAs on the autophagy pathway in CVDs, while Tables 7, 8 present the pathological conditions and models employed to delineate the functions of lncRNAs in vascular cells and cardiomyocytes, respectively.

### 4.8 Circular RNAs modulating autophagy in vascular and cardiac tissues

CircRNAs are lncRNAs that are covalently closed to form a loop without the 5′ and 3′ polarities (Hombach and Kretz, 2016). This structure makes them more stable than linear RNAs (Chen and Lu, 2021). The functions of circRNAs include sponging for miRNAs and proteins, acting as scaffolds for RNA binding proteins, and regulating splicing and translation (Lu and Thum, 2019). This class of ncRNAs was discovered most recently, and its involvement in CVDs is a rapidly emerging and expanding research area (Altesha et al., 2019; Xie et al., 2024). For example, hsa_circ_0001402 has been found to bind miR-183-5p, which in turn promotes proliferation and migration of VSMCs by targeting FKBP prolyl isomerase like (FKBPL) while inhibiting autophagy targeting BECLIN1. Therefore, overexpression of hsa_circ_0001402 alleviates phenotypic switching in VSMCs (Lin et al., 2024) even as circ_0002331 promotes proliferation while repressing apoptosis, autophagy, and inflammation in dysfunctional ECs. Mechanistically, circ_0002331 positively regulates cyclin D2 (CCND2) mRNA stability by interacting with ELAV like RNA binding protein 1 (ELAVL1) (Chen and Yu, 2024). CCND is a protein belonging to the cyclin family that promotes cell proliferation and reduces apoptosis in HUVECs (Wu et al., 2016). Another circRNA that regulates autophagy is hsa_circ_0030042, which is downregulated in coronary heart disease; hsa_circ_0030042 obstructs eIF4A3 recruitment to BECLIN1 and FOXO1 mRNAs, thereby inhibiting abnormal autophagy in HUVECs. Moreover, upregulation of hsa_circ_0030042 in ApoE^−/−^ mice fed a high-fat diet was found to reduce autophagy (Yu et al., 2021). In primary mouse neonatal cardiomyocytes (MNCs) treated with H_2_O_2_, circ-HIPK2 impacts autophagy by sponging miR-485-5p; this interaction blocks miRNA activity and permits upregulation of ATG10, with consequent enhancement of autophagy, suppression of apoptosis, and acceleration of MNC proliferation (Zhou et al., 2020). Another circRNA involved in autophagy regulation is circHIPK3; using cardiomyocytes, Qiu et al. (2021) demonstrated that it acts as an endogenous miR-20b-5p sponge that permits expression of ATG7 during myocardial and H/R injuries. Figure 3 summarizes the impacts of circRNAs on the autophagy pathway in CVDs. Table 9 summarizes the studies reported herein, including the pathological conditions and models used.

**TABLE 9:** Circular RNAs regulating autophagy in CVDs.

Circular RNAs	Condition/Disease	Models	Non-coding RNA roles	References
circ_0002331	AS	Human umbilical vein ECs treated with ox-LDL	Inhibits autophagy through stabilization of CCND2 mRNA	Chen and Yu (2024)
circ-HIPK2	Myocardial injury	Primary mouse neonatal CMs treated with H_2_O_2_	Promotes autophagy by sponging miR-485-5p to induce ATG10	Zhou et al. (2020)
circ-HIPK3	Myocardial I/R injury	I/R injury mouse model and H/R-treated neonatal mouse ventricular CMs	Promotes autophagy by sponging miR-20b-5p to induce ATG7	Qiu et al. (2021)
Has_circ_0030042	AS/coronary heart disease	Human umbilical vein ECs treated with ox-LDL and high-fat-diet-fed ApoE^−/−^ mouse	Sponges eIF4A3 and blocks its recruitment to BECLIN1 and FOXO1 mRNAs	Yu et al. (2021)
hsa_circ_0001402	Neointimal hyperplasia	Neointimal hyperplasia mouse model and human aortic SMCs	Promotes autophagy by sponging miR-183-5p to induce BECLIN1	Lin et al. (2024)

ECs: endothelial cells, CMs: cardiomyocytes, SMCs: smooth muscle cells, ox-LDL: oxidized low-density lipoprotein, H/R: hypoxia/reoxygenation, I/R: ischemia/reperfusion, AS: atherosclerosis.

### 4.9 Preclinical models demonstrate the significance of ncRNAs as drugs for treating CVDs

RNA-based therapeutics are classified into various types as small interference RNA (siRNA)-based therapeutics, miRNA-based therapeutics, antisense oligonucleotides (ASOs), RNA aptamers, ribozymes, and mRNA-based therapeutics (Chatterjee et al., 2023). Most RNA-based drugs belong to the siRNA and ASO classes (Chatterjee et al., 2023). SiRNAs are dsRNA molecules having lengths of approximately 18–25 nucleotides and structures that can be directly recognized by and loaded onto the RISC complex. Therefore, siRNAs use cellular machinery to downmodulate specific RNA molecules (Zhu et al., 2022; Chatterjee et al., 2023; Zhang and Zhang, 2023). The newest class of miRNA-based therapeutics is currently in phase II or III of clinical trials (Nappi, 2024) and includes miRNA mimics and inhibitors. MiRNAs mimics are synthetic RNAs that can mimic the upregulation of specific miRNAs to simultaneously act on different targets, similar to endogenous miRNAs. However, miRNA inhibitors (also known as anti-miRs or antagomiRs) are a class of single-stranded RNA molecules that can bind endogenous miRNAs simulating the activities of ceRNAs to prevent miRNA activity (Chatterjee et al., 2023). ASOs are short single-stranded DNA or RNA sequences with lengths of 12–24 nucleotides that are designed to target sequence-specific RNAs. ASOs can degrade a target mRNA or lncRNA through cleavage of RNA by RNase H; moreover, ASOs can block proteins that bind RNAs to inhibit mRNA translation or can enter the nucleus to modulate splicing (Zhu et al., 2022; Zhang and Zhang, 2023). ASOs also act as anti-miRs to target and inactivate mature miRNA forms (Chatterjee et al., 2023). Before developing a drug, it is imperative to comprehend its molecular activities and test its efficacy. This necessitates the use of preclinical models, which can be divided into *in vitro* models comprising two- or three-dimensional cell cultures and single cell assays, *ex vivo* models, *in vivo* models, and bioinformatics-based models.

*Bioinformatics-based models.* With the advent of artificial intelligence (AI), models based on bioinformatics appear promising but need large amounts of data obtained from real experiments for training. Therefore, these are often based on *in vitro, ex vivo,* and *in vivo* models. Bioinformatics approaches have recently been employed to simulate cardiomyocyte calcium handling, which is crucial for the development of cardiac arrhythmias (Sutanto et al., 2020). Potassium current is known to be involved in cardiac ventricular repolarisation; thus, Meier et al. (2023) developed an *in silico* model considering Kv 11.1 ion-channel trafficking and gating to reveal a complex temporal regulation of cardiac electrophysiology based on temperature, hypokalemia, and dofetilide. These efforts are merely a few examples as several other works published within the last 5 years have demonstrated the significance of bioinformatics approaches in investigating the effects of electromechanical coupling and pharmacological actions on human ventricular electrophysiology (Margara et al., 2021). Bioinformatics analyses have also been conducted to comprehend thrombus formation through modelling of fluid dynamics in the left atrium (Boyle et al., 2021) and to enhance valve design for optimising structural performance (Whiting et al., 2022). With regard to RNAs, bioinformatics and AI in particular could enhance our understanding of RNA–RNA or RNA–protein interactions by elucidating the molecular structures of these molecules. The 3D structure of RNA is particularly crucial for lncRNAs as it enables their specific interactions with proteins (e.g. the lncRNA growth-arrest-specific 5 regulates cell survival through distinct structural modules) (Frank et al., 2020). Similarly, the 3′-UTR shape of the coding genes permits or prevents (if the structure masks the binding site) miRNA binding (Yang et al., 2020).

*In vitro models. In vitro* models are cheaper, more accessible, and simpler than *ex vivo* and *in vivo* models; hence, they can also be used in high-throughput analyses. *In vitro* analyses show the contributions of single-cell types but do not account for the interactions between cells. To overcome this problem, cell cultures are performed with different cells but deducing the cause–effect relationships could become complicated as the outcomes could be influenced by more than one element. One solution to this problem that can also be applied to tissues is single-cell analysis based on newly developed transcriptomic techniques (Alessio et al., 2020). These techniques are widely used to understand the composition of heart tissue and to evaluate changes in cell compositions or single-cell gene expressions in response to different pathological states (Yamada and Nomura, 2020; Wang et al., 2022; Miranda et al., 2023). The development of induced pluripotent stem cells (iPSCs) has enabled studies on the various aspects of CVDs. In fact, they allow the generation of virtually all cell types harbouring pathological mutations, thereby eliminating the need for continuous tissue sampling to investigate pathological conditions. Alternatively, they may serve as the foundation for therapeutic interventions, such as myocyte substitution in MI. Notably, cellular reprogramming, pluripotency, cardiac differentiation, and maturation are regulated by ncRNAs (Hunkler et al., 2022). Therefore, a comprehensive understanding of the biological functions of ncRNAs associated with these processes can significantly enhance the quality and quantity of stem cells as well as their respective derivatives, paving the path for safer and more efficient cell therapy approaches in heart failure. *In vitro* models provide more realistic representations if the cells are cultured in 3D structures. Several approaches have been employed to generate 3D human cardiac constructs from iPSCs by using scaffolds composed of hydrogels (Williams et al., 2021), bioprinters comprising cell suspensions as part of their inks (Lippi et al., 2020), cells seeded onto decellularized scaffolds (Tenreiro et al., 2021), or organoid production (Zhao et al., 2021a). Thus, different cells and even iPSCs can be integrated on a chip to reduce the sample volumes required for drug tests (Skardal et al., 2020). With regard to ncRNAs, the stiffness of the 3D structure is particularly crucial in such experiments; in fact, ncRNAs play an important role in modulating the conformation of the ECM, thereby influencing the phenotypes of the interacting cells (Akbari Dilmaghnai et al., 2021). For example, fibrosis characterized by excessive collagen production and ECM accumulation is associated with heart failure, valve tinkering, and other cardiovascular conditions. Recently, the lncRNA Airn has been found to have antifibrotic activity; it prevents ubiquitination-dependent degradation of insulin-like growth factor 2 mRNA-binding protein 2 (IMP2) and protects p53 mRNA from degradation, leading to cardiac fibroblast cell-cycle arrest (Peng et al., 2022). Consequently, variations in the outcomes obtained for various experimental conditions of *in vitro* models may be attributed to alterations in the stiffness of the ECM. For instance, the stiffnesses of plastics used in two-dimensional cultures are significantly different from those of hydrogels suitable for three-dimensional cultures.

*Ex vivo models.* In contrast to *in vitro* models, *ex vivo* preclinical models, particularly those used in perfusion experiments involving the heart, are not well-suited for analyzing ncRNAs. These models are based on living tissues maintained in an artificial environment outside the body. This artificial setting can cause alterations in RNA stability, potentially resulting in degradation or structural changes in response to the altered physiological conditions. As elucidated previously, these structural changes are crucial for the activities of the lncRNAs. Otherwise, artificial conditions can induce non-canonical ncRNA expressions that can lead to misinterpretations or misguided development of therapeutic approaches associated with RNAs. However, it is indisputable that *ex vivo* models are particularly useful for fluid dynamic studies, analyses on contractile functions associated with ischemia and hypoxia, and studying rhythm disorders (van Doorn et al., 2024).

*Animal models.* Small animals (*Drosophila*, zebrafish, *Xenopus*, mice, and rats), medium-sized animals (guinea pigs, rabbits, and cats), and large animals (dogs, pigs, sheep, and non-human primates) represent one of the cornerstones of preclinical research efforts. However, animal models have specific limitations. They possess distinct genetic backgrounds compared to humans, and the induction of various pathologies often results in incomplete recapitulation of the pathological progression observed in humans. For instance, the aging process in mice is faster than in humans. Consequently, the accumulation of age-related events that transpire in humans may not be concomitant to other alterations in mice. As a general principle, larger animals have greater biological resemblance to humans, with better translational applicability at increased costs. Recently, several lncRNAs were associated with the development of CVDs using animal models. Kcnq1ot1 is a type of lncRNA whose expression is increased in the myocardium infracted zones of rats whose left anterior descending coronary arteries (LAD) were ligated to induce MI. It was demonstrated that Kcnq1ot1 inhibits the interaction of miR-466-5p with the transcription factor TEA domain family member 1 (TEAD1) to trigger cardiomyocyte injury (Liao et al., 2020). Previously, we have underscored the importance of potassium channels in ventricular repolarization. From a genomic standpoint, it is particularly interesting that an ncRNA involved in cardiomyocyte injury is synthesized from the reverse strand of the potassium voltage-gated channel subfamily Q member 1 coding gene, where mutations in this gene are associated with inherited arrhythmias. The same experimental setup was employed to demonstrate the activity of the lncRNA MIRT2 on miR-764, where it mitigates the inhibitory effect of miR-764 on 3-phosphoinositide-dependent kinase 1 (PDK1). PDK1 is situated within the mitochondrial matrix and plays a crucial role in cellular metabolism and apoptosis (De Rosa et al., 2021). LAD ligation in rats is a widely employed technique to investigate the functions of ncRNAs in MI and has also been used to demonstrate the ability of the lncRNA FGF9-associated factor (FAF) to sponge for miR-185-5p (Gu et al., 2022). P21 activated kinase 2 (PAK2) has a cardioprotective role in several CVDs (Binder et al., 2022; Xu et al., 2022); therefore, avoiding its downmodulation through sponging of miR-185-5p that can target PAK2 has beneficial effects on hypoxia/ischemia-induced pyroptosis (Gu et al., 2022).

Instead of relying solely on rats, it is feasible to replicate the previously described methodology for rats to simulate MI in mice. This approach was used to demonstrate that the lncRNA nuclear paraspeckle assembly transcript 1 (NEAT1) binds to miR-450b-5p (Yu et al., 2024). It is not surprising that NEAT1 interacts with miRNAs because it is involved in several RNA processes, such as mRNA polyadenylation, retention in the nucleus, or pri-miRNA processing (Knutsen et al., 2022). We previously discussed the role of the lncRNA 2810403D21Rik/AK007586/Mirf in regulating autophagy by modulating the activity of miR-26a. It is widely recognized that miRNAs do not have single targets. Su et al. (2020) demonstrated that miR-26a may also be involved in the modulation of cardiac apoptosis. Notably, miR-26a regulates BCL2 antagonist/killer 1 (BAK1), and its sequestration by Mirf stimulates apoptosis through the release of mitochondrial cytochrome C. The lncRNA FAF interacts with miR-185-5p in hypoxia/ischemia-induced cardiomyocytes. This interaction allows upregulation of PAK2 to protect the heart (Gu et al., 2022). Another lncRNA that sponges miRNAs and is associated with I/R myocardial injury is AK020546; it avoids the action of miR-350-3p on Erb-B2 receptor tyrosine kinase 3 that activates the RAC (Rho family)-alpha serine/threonine-protein kinase (AKT) to inhibit BCL2 associated agonist of cell death/BCL2 (Bad/Bcl2) (Zhang et al., 2021c). Therefore, activation of the lncRNA AK020546 can protect against myocardial apoptosis.

Additional intriguing evidence on the interactions between lncRNAs and miRNAs has been proposed by Niu et al. (2020). These authors demonstrated that Opa-interacting protein 5-antisense transcript 1 (OIP5-AS1) interacts with miR-29a to decrease the apoptosis induced by myocardial I/R injury (Niu et al., 2020). Bai et al. (2021) conducted research on the functions of the lncRNA MIAT in the mouse model of MI; their findings reveal that MIAT silencing induces reductions of the translocation protein (TSPO) at both the mRNA and protein levels in MI. Indeed, such silencing prevents mitochondrial permeability transition pore opening, thereby safeguarding the integrity of the mitochondrial membrane from hypoxic damage (Bai et al., 2021).

These studies emphasize the importance of ncRNAs in intracellular communications, particularly those between the nucleus and mitochondria. These organelles are involved in not only metabolic cell regulation but also the regulation of cell survival and gene expression modulation through epigenetic mechanisms. Notably, acetyl-CoA is synthesized in the mitochondria by metabolizing fatty acids and plays a pivotal role in the epigenetic modifications of histones (He et al., 2023). Metabolic alterations in mice with MI induced by LAD ligation can be modulated through another lncRNA called the cardiomyocyte pyroptosis-associated (CPAL). Instead of using a mechanism based on miRNA interaction, CPAL directly interacts with nuclear factor kappa B (NF-κB) to induce its activation through phosphorylation (Li et al., 2023). NF-κB plays important roles in CVDs, atherosclerosis, and diabetes; it is responsible for the regulation of genes involved in inflammation, immune responses, and fibrosis. Several therapeutic agents used to treat CVDs and diabetes, such as pimobendan and sodium–glucose cotransporter 2 inhibitors, inhibit NF-κB activation. Therefore, the possibility of using an siRNA to downmodulate the expression of the lncRNA CPAL (an NF-κB activator) is a viable alternative for modulating several detrimental aspects of CVDs.

Mitochondrial shapes and metabolic states are strictly related (Chemello et al., 2019), where mitochondrial shaping is regulated by fusion or fission proteins. Interestingly, dynamin-related protein 1 (DRP1) promotes mitochondrial fission (Gao et al., 2020) that can be modulated by the interactions between DRP1 and OIP5-AS1. These interactions prevent cellular apoptosis during MI (Niu et al., 2024). Several other lncRNAs have been demonstrated to interact with proteins and modulate different phenomena associated with MI. For instance, the lncRNA cardiac conduction regulatory RNA (CCRR) binds to the toll-like receptors 2 and 4 (TLR2 and TLR4) to inhibit inflammation during MI (Wang et al., 2024b). Additionally, the lncRNA small nucleolar RNA host gene 1 (SNHG1) interacts with phosphatase and tensin homolog (PTEN) to induce its degradation (Li et al., 2021b). The upregulation of SNHG1 has beneficial effects as PTEN degradation activates the phosphoinositide 3-kinase (PI3K)/protein kinase B (AKT) pathway to promote cardiomyocyte proliferation. Furthermore, the lncRNA DACH1 directly binds to protein phosphatase 1 catalytic subunit alpha (PP1A) to limit its dephosphorylation activity. This process is crucial for cardiac repair and regeneration after MI since the interaction of PP1A with lncRNA DACH1 permits phosphorylation of yes-associated protein 1 (YAP1), limiting its nuclear translocation (Cai et al., 2020). This exemplifies a sophisticated mechanism of cell-cycle regulation through modulation of the Hippo/YAP1 pathway by lncRNAs. The lncRNA cardiac fibroblast-associated transcript (Cfast) also interacts with a protein to modulate cardiac fibrosis. Here, Cfast interacts with coactosin-like 1 (COTL1) (Zhang et al., 2021a), which then interacts with TNF receptor associated protein 1 (TRAP1) to promote formation of the SMAD2/4 complex leading to fibrosis (Wang et al., 2015). Therefore, blocking this interaction through COTL1 sequestration on Cfast can abrogate fibrosis. Interestingly, TRAP1 is also involved in several other cellular activities, including modulation of cellular metabolism (Amoroso et al., 2014). Therefore, Cfast may be another example of an RNA involved in communication between the nucleus and mitochondria. The associations between lncRNAs and proteins can impact the assembly of a “transcriptosome complex” to favor or avoid gene transcription; here, the latter is exemplified by the interactions between the lncRNA scaffold attachment factor B interacting (SAIL) and SAFB (Luo et al., 2021). SAIL is downregulated in fibrotic cardiac tissues and ameliorates fibrosis when upregulated. Luo et al. (2021) demonstrated that SAIL interacts with SAFB and limits its availability to form complexes to permit transcription of profibrotic genes. The cardiac ischemia reperfusion associated Ku70 interacting lncRNA (CIRKIL) is another example that modulates the effects of myocardial I/R through interaction with a specific protein; CIRKIL is upregulated in the I/R myocardium and leads to a decrease in the nuclear translocation of Ku70 (Xiao et al., 2022), a DNA repair subunit protein that binds to DNA double-strand breaks and facilitates repair via the non-homologous end-joining repair pathway. During I/R, there is nuclear translocation of the BCL2-associated transcription factor 1 (Bclaf1) by the cardiac injury-related Bclaf1-inhibiting lncRNA (CIRBIL); once translocated into the nucleus, Bclaf1 promotes transcription of TP53 (p53) to suppress the expression of CIRBIL and activate apoptosis in the cardiomyocytes (Zhang et al., 2021b).

CIRBIL levels are reduced in I/R hearts; consequently, therapeutic approaches to reverse cardiomyocyte death based on modulation of CIRBIL expression must be based on upregulation of CIRBIL. However, this approach may pose challenges as cells require transfection with a plasmid that exceeds the dimensions of conventional siRNAs used for gene silencing. Furthermore, transcription of lncRNAs should be precisely regulated to avoid toxicity associated with excess concentrations, which can lead to binding with non-canonical proteins. In contrast to CIRBIL, cardiac ischemia reperfusion associated p53 interacting lncRNA (CIRPIL) inhibits apoptosis of cardiomyocytes exposed to anoxia. Jiang et al. (2022) demonstrated that CIRPIL operates in the direction opposite to the lncRNA CIRBIL; notably, CIRPIL sequesters p53 in the cytoplasm and prevents its translocation into the nucleus. This mechanism inhibits the activation of apoptotic genes and prevents cardiomyocyte death. P53 is also central to the function of lncRNA-6395 and binds to it to avoid p53 ubiquitination and degradation. Therefore, upregulation of lncRNA-6395 during I/R injury promotes cardiomyocyte apoptosis through stabilisation of p53 that permits its nuclear translocation (Zhan et al., 2022). The lncRNA HOX transcript antisense RNA (Hotair) is upregulated in response to I/R stimuli; Hotair exerts protective effects on oxidative stress and cardiac myocyte apoptosis by binding to the enhancer of zeste homolog 2 (EZH2) to modulate the expression of miR-451 involved in the regulation of myocyte apoptosis and oxidative stress (Meng et al., 2020). The action mechanism of Hotair is unique and seems to be a combination of two mechanisms described earlier, namely, lncRNA–protein and lncRNA–miRNA interactions. Hotair interacts with a protein, but this interaction modulates the expression of a miRNA, thereby regulating the expression of another protein. Yu et al. (2023b) showed the importance of the lncRNA myeloid RNA regulator of Bim-induced death (Morrbid) to protect the heart from acute MI; the authors showed a correlation with serpin family E member 1 (SERPINE1) without demonstrating a direct interaction with the lncRNA.

By leveraging a unique cardiac characteristic, Fu et al. (2022) discovered genes associated with cardiac regeneration and suggested the potential for activating this process, which is diminished in the adult heart. Notably, mammalian cardiomyocytes are capable of regeneration for a limited period following birth. Apical resection (AR by exposure of the left ventricular apex and surgical amputation) in 1-day-old mice was shown to trigger a regenerative response that restored the damaged heart to its normal anatomical and functional state (Porrello et al., 2011). Fu et al. (2022) leveraged AR to identify the lncRNA natriuretic peptide A antisense RNA 1 (NAPPA-AS1) that exhibited inhibitory effects on cardiomyocyte proliferation. NAPPA-AS1 expression is downregulated during cardiomyocyte regeneration following AR activation, which enables regeneration. NAPPA-AS1 interacts with the protein splicing factor SFPQ that is involved in DNA repair; consequently, the absence of this lncRNA during cardiomyocyte proliferation allows SFPQ functions, i.e. DNA damage prevention and subsequent cardiomyocyte proliferation. A unique study conducted by Aghagolzadeh et al. (2023) on mouse heart aimed to evaluate gene expressions at the single-cell level during MI; this study is notable for its innovative approach that enables the identification of cell composition changes during the development of pathologies. Furthermore, since lncRNAs exhibit more cell-type-specific expressions compared to coding RNAs, it is crucial to ascertain which cells express specific lncRNAs. Notably, Aghagolzadeh et al. (2023) demonstrated that cardiac cell identity is solely defined by the expressions of lncRNAs in single-cell experiments. Additionally, they identified a novel lncRNA named fibrogenic LOX-locus enhancer RNA (FIXER) that interacts with the protein chromobox 4 (CBX4) to guide gene expression regulators to the promoter of the RUNX family transcription factor 1 (RUNX1). This interaction induces the expression of RUNX1, which positively regulates the expressions of fibrogenic genes. From a therapeutic perspective, downregulation of FIXER has been shown to limit fibrosis and improve heart function.

In MI, the fibroblasts and cells involved in vascular system development must also be targeted for replacement in addition to cardiomyocytes. Atherosclerosis and thrombosis affect the functionality of tissues supplied by arteries and veins, respectively, and can impair vascular function. Angiogenesis is crucial for vascular development, and neovascularization is the bodily response to ischemia. Identifying lncRNAs that stimulate neovascularization holds potential for the treatment of MI. Wu et al. (2021b) addressed this challenge by comparing the cardioprotective effects of embryonic stem-cell-derived MSCs (ES-MSCs) with human bone-marrow-derived MSCs (BM-MSCs); they identified lncRNAs upregulated in ES-MSCs compared to BM-MSCs and found that the stem-cell-derived angiogenic lncRNA (SCDAL) induces the expression of growth differentiation factor 6 (GDF6), which promotes endothelial angiogenesis. Notably, the activation of GDF6 is mediated by recruitment of the SWI/SNF chromatin-remodeling protein SNF5 to the GDF6 promoter through SCDAL (Wu et al., 2021b). MSCs can be modified to inhibit the expression of the lncRNA small nucleolar host gene 12 (SNHG12), which significantly enhances their effects in activating the PI3K/AKT/mTOR signaling pathway both *in vitro* and *in vivo* (Li et al., 2019a). Therefore, modified MSCs may ameliorate I/R injuries via the PI3K/AKT/mTOR signaling pathway. Another lncRNA that modulates the AMPK/mTOR pathway during ischemic stroke is AC136007.2. Liu et al. (2021) demonstrated that intraventricular lncRNA AC136007.2 administration reduced cerebral infarction and brain edema in a rat model of ischemic stroke. Several other lncRNAs are involved in atherosclerosis, heart failure, arrhythmia, cardiomyopathies, and diabetic cardiomyopathy, as described by Xie et al. (2025).

*Large animal models.* Compared to rodents, pigs have many advantages as animal models given that their anatomy, physiology, metabolism, and immune system are more similar to those of humans; thus, pigs were used to demonstrate that the lncRNA rhabdomyosarcoma 2-associated transcript (RMST) acts as a competitive endogenous RNA of miR-24-3p. Ma et al. (2023) evaluated the possibility of modulating RMST to alleviate cardiac fibrosis and improve cardiac function in a porcine model of MI; they demonstrated that the downmodulation of RMST improves fibrosis and cardiac functions after MI.

### 4.10 Application of ncRNAs in the treatment of CVDs

Although ncRNAs present promising opportunities in medicine, there are concerns regarding potential adverse effects and undesirable outcomes caused by off-targeting. Indeed, single-stranded or double-stranded RNAs utilized for therapeutic purposes may be recognized by the immune system as viruses, potentially triggering undesirable immune responses. In the case of siRNAs, immune stimulation reflects special siRNA sequences, siRNA delivery vehicle types, and RNAi-directed RNA cleavage products. Therefore, siRNA sequence modulation can be used to modulate immune responses. It is known that avoiding immunostimulatory motifs, such as 5′-UGU-3′, 5′-UGUGU-3′ (Judge et al., 2005), and 5′-GUCCUUCAA-3′ (Hornung et al., 2005), can limit immunostimulation. Additionally, nucleotide chemical modifications, such as 2′-O-methyl purines, 2′-fluoropyrimidines, 2-thiouracil or pseudouracil, PS linkage modifications, 5-methyl-C, and N6-methyl-A pseudouridine, can prevent immune activation (Meng and Lu, 2017). RNA-based therapies still have challenges related to delivery. These are simpler in the cases of cells that can be purified from patients before being *in vitro* cultivated, treated, and reimplanted in patients (e.g. blood cells), but reaching specific tissues like the heart or vessels that are broadly distributed in the body is more complicated. In fact, treatment through the blood system is often affected by the filtration ability of the liver. These topics have been recently reviewed by Androsavich (2024) and Nappi (2024); therefore, in this review, we focus on RNA-based therapeutics to treat CVDs. As discussed in the previous sections, ncRNAs are involved in several processes of CVDs, and the modulation of autophagy by ncRNAs is a valid alternative to classical approaches. Current RNA-based drug candidates aim to modulate aspects associated with the development of hypertension and vascular permeability. For example, they aim to correct dyslipidemia to limit atherosclerosis (Burvill et al., 2024; National Library of Medicine, 2024; Nissen et al., 2024a, 2024b) or prevent angiotensin II synthesis to modulate hypertension (Bakris et al., 2024; National Library of Medicine, 2024). It is known that overexpression of miR-26a attenuates ischemic-stress-induced cell death by activating autophagy through targeting Usp15 (Liang et al., 2020). Consequently, upregulation of miR-26a via miRNA mimics may confer beneficial effects on ischemic stress. However, miR-26a and its corresponding miRNA mimic can be captured by lncRNAs that are upregulated in MI mice. As discussed previously, the lncRNA 2810403D21Rik/AK007586/Mirf acts as a sponge for miR-26a, thereby inhibiting its beneficial effects. From a therapeutic standpoint, the previously described interaction underscores the importance of comprehending the expressions of lncRNAs or circRNAs that can influence the activities of therapeutic miRNAs.

The translation of basic discoveries into treatments for human diseases (via the “bench-to-bedside” approach) is a lengthy and risky process. It has been estimated that discovery to approval of a new drug could take more than 13 years and could fail in 99.9% of cases. Only 0.1% of new drug candidates from preclinical studies are approved by regulatory authorities. This phenomenon is known as the valley of death (Seyhan, 2019; Everts and Drew, 2024). Currently, the only RNA-based therapy approved by the United States Food and Drug Administration (USFDA) or European Medicines Agency (EMA) for treating CVDs is Leqvio^®^, which is also known as inclisiran or KJX839 (Novartis Pharmaceuticals). Leqvio is indicated for lowering the concentration of low-density lipoprotein cholesterol (LDL-C) in adults affected by heterozygous familial hypercholesterolemia or clinical atherosclerotic CVD (EMA/FDA 2021). Inclisiran is a double-stranded siRNA targeting proprotein convertase subtilisin kexin type 9 (PCSK9) mRNA; PCSK9 downregulation increases LDL-C receptor recycling and expression on the hepatocyte cell surface. This increases LDL-C uptake to lower blood LDL levels (U.S. Food and Drug Administration, 2024). There are several ongoing clinical trials in phase 3 or 4 to extend the indications and usage of Leqvio. Olpasiran (Amgen), SLN360 (Silence Therapeutics), and Pelacarsen (Novartis Pharmaceuticals) are some of the new drug candidates that are now in clinical experimentation for the treatment of atherosclerosis by targeting the mRNA of apolipoprotein (a) to lower lipoprotein(a). Lowering the amount of lipoprotein(a) could reduce the risk of CVD. Furthermore, Zilebesiran (Alnylam Pharmaceuticals) and ADX-850 (ADARx pharmaceutical, Inc.) have been developed to act on the angiotensin system to treat hypertension (National Library of Medicine, 2024). Currently, there is only one RNA-based drug candidate targeting a ncRNA; it is an ASO named CDR132L that was designed to bind miR-132-3p; it was developed to treat heart failure because blocking the pathological overexpression of miR-132-3p reduces heart remodeling and improves cardiac functions in heart-failure patients (National Library of Medicine, 2024). It has been initially assessed for antimiR-132 therapeutic efficacy, pharmacokinetic efficacy, and safety in a large animal (pig) model of heart failure (Foinquinos et al., 2020). Subsequently, CDR132L has been assessed in phase 1 (Täubel et al., 2021) and then phase 2 (Bauersachs et al., 2024) trials. Table 10 presents a summary of the ongoing clinical trials aimed at developing effective treatments for CVDs.

**TABLE 10:** Ongoing clinical trials for CVDs.

Candidate	Sponsor	Type	Target	Condition	Phase	ID NCT	Study starting year	References
Olpasiran AMG 890	Amgen	siRNA	Apolipoprotein (a)	ASCVD	3	NCT05581303	2022	Burvill et al. (2024)
CDR132L	Cardior Pharmaceuticals GmbH	ASO	miR-132-3p	Heart failure	2	NCT05953831	2024	
				Heart failure, myocardial infarction	2	NCT05350969	2022	Bauersachs et al. (2024)
Zerlasiran SLN360	Silence Therapeutics PLC	siRNA	Apolipoprotein (a)	ASCVD	2	NCT05537571	2023	Nissen et al. (2024a), Nissen et al. (2024b)
ADX-850	ADARx Pharmaceutical, Inc.	siRNA	Hypoxanthine phosphoribosyltransferase	Hypertension	1	NCT06205628	2024	
Zilebesiran ALN-AGT01	Alnylam Pharmaceuticals	siRNA	Angiotensinogen	Hypertension	2	NCT04936035	2021	Bakris et al. (2024)
					2	NCT05103332	2021	
					2	NCT06272487	2024	
					2	NCT06423352	2024	
Pelacarsen TQJ230	Novartis Pharmaceuticals	ASO	Apolipoprotein (a)	ASCVD	3	NCT04023552	2019	Burvill et al. (2024)
					3	NCT06267560	2024	
					3	NCT05900141	2023	
Inclisiran Leqvio^®^ KJX839	Novartis Pharmaceuticals	siRNA	Proprotein convertase subtilisin/kexin type 9 (PCSK9)	ASCVD	4	NCT06501443	2024	
	Icahn School of Medicine at Mount Sinai			ASCVD	3	NCT06494501	2024	
	Novartis Pharmaceuticals			ASCVD	4	NCT06431763	2024	
	Jose Seijas Amigo			Ischemic heart disease, acute coronary syndrome	4	NCT06421363	2024	
	Novartis Pharmaceuticals			Atherosclerosis, myocardial infarction	4	NCT06372925	2024	
	University of Louisville			Atherosclerosis, coronary artery disease	4	NCT06280976	2024	
	Novartis Pharmaceuticals			ASCVD	3	NCT05030428	2021	

ASCVD: Atherosclerotic cardiovascular disease. Source: https://clinicaltrials.gov/

## 5 Meta-analysis: identification of genes implicated in CVDs

**FIGURE 4:**
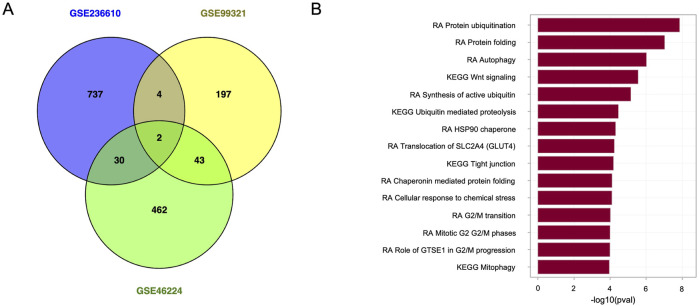
Representation of differentially expressed genes (DEGs). **(A)** Venn diagram of the DEGs reported in different studies. **(B)** Pathway enrichment analysis of the DEGs common to at least two studies. RA, Reactome database; KEGG, Kyoto encyclopedia of genes and genomes pathway database.

**FIGURE 5:**
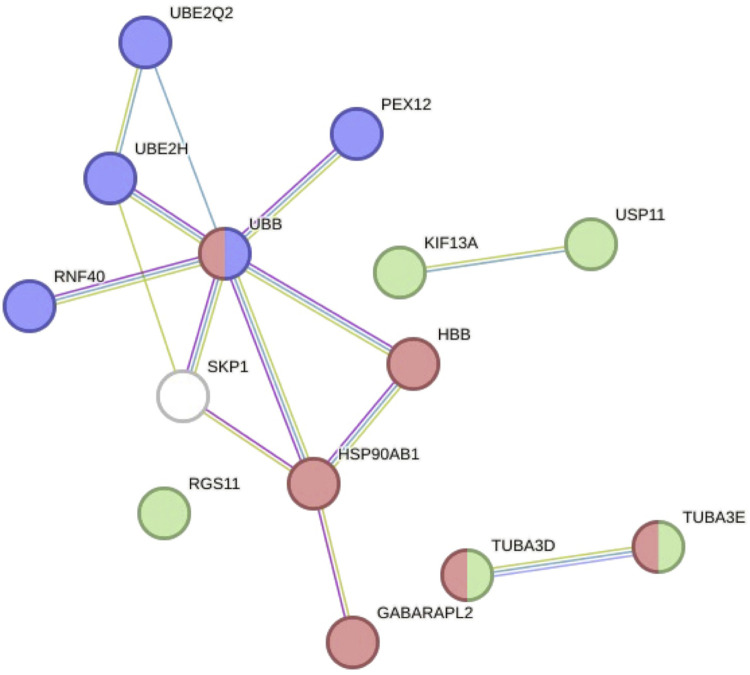
Network of DEGs shared by at least two studies and related to autophagy. The DEGs shared by at least two studies code for proteins related with autophagy as well as proteins that interact with each other to form a network described by two big clusters—one associated with ubiquitination (blue nodes) and another associated with autophagy and chaperone-mediated protein folding (red and green nodes, respectively)—according to the Reactome database. The light-green edges represent interactions retrieved by text mining, violet ones represent experimentally identified interactions, and light-blue ones represent interactions sourced from curated databases.

Gene expression data from coronary plaques (GSE236610; Widlansky et al., 2023) as well as left ventricles of patients with pediatric idiopathic dilated cardiomyopathy (GSE99321; Tatman et al., 2017) and heart failure (GSE46224; Yang et al., 2014) were retrieved using the parameters described in the methods section. A total of 773 differentially expressed genes (DEGs) were found by comparing samples from stable CAD with those from acute coronary syndrome (Supplementary Table S2), 274 DEGs were obtained by comparing controls and patients with idiopathic dilated cardiomyopathy (Supplementary Table S2), and 555 DEGs were noted by comparing controls and heart failure (Supplementary Table S2). Interestingly, if we simply compare these lists of DEGs, two genes were common among all studies (Figure 4A): Ena/vasodilator-stimulated phosphoprotein-like (*EVL*) and GABA type A receptor-associated protein like 2 (*GABARAPL2*). Both genes are important for autophagy. *EVL* is a member of the Ena/VASP protein family that functions as a highly efficient actin elongation factor; it was demonstrated that *EVL* is important for autophagosome formation and trafficking and that it colocalizes with MAP1LC3/LC3 so that mammalian ATG9A forms a ring-like structure around EVL-LC3 (Li et al., 2024). *GABARAPL2* is a member of the ATG8 family that is involved in autophagy and is associated with autophagosomes (Jacquet et al., 2021; Chen et al., 2024b). Considering the functions of the genes shared by at least two datasets, we showed that these genes are involved in pathways that are directly related to autophagy (Figure 4B). The genes associated with the pathways represented in Figure 4B are as follows: *PEX12*, *UBB*, *UBE2H*, *UBE2Q2*, and *RNF40* for protein ubiquitination; *TUBA3E*, *TUBA3D*, *KIF13A*, *USP11*, and *RGS11* for protein folding; *TUBA3E*, *GABARAPL2*, *TUBA3D*, *HBB*, and *HSP90AB1* for autophagy; *UBB*, *UBH*, and *UBE2Q2* for synthesis of active ubiquitin; *UBB*, *UBH*, *UBE2Q2*, and *SKP1* for ubiquitin-mediated proteolysis; *TUBA3E*, *TBA3D*, and *HSP90AB1* for HSP90 chaperone cycle. Interestingly, proteins coded from genes associated with previously cited pathways are strictly related; they essentially form two groups of proteins, namely those associated with ubiquitination and those associated with autophagy and chaperone-mediated protein folding (Figure 5).

## 6 Conclusions and future perspectives

The present review considers recently published works demonstrating the importance of autophagy in aging and CVDs. Cardiovascular aging contributes significantly to the pathogenesis of age-related CVDs. Oxidative stress, mitochondrial dysfunction, and inflammation are some of the hallmarks of aging, and their involvement in CVDs underscores their crucial roles in the development of these conditions. Oxidative stress is regulated by reactive oxygen species and reactive nitrogen species, which are among the primary intracellular signal transducers that sustain autophagy. Conversely, dysfunctional mitochondria are eliminated through mitophagy. Notably, as the myocardium is a highly oxidative metabolic tissue, mitochondria play central roles in maintaining optimal cardiac functions. Alterations to or impaired elimination of non-functional mitochondria may pose significant challenges to myocardial functions. Furthermore, autophagy exerts significant effects on the induction and modulation of inflammatory responses (Pang et al., 2022). Consequently, processes that are directly associated with aging and the development of CVDs are either modulated by or modulate autophagy. However, the initial processes associated with aging that initiate the development of CVDs remain poorly understood.

Autophagy modulation is regarded as a promising mechanism of programmed cell death that has the potential to prevent and treat a wide range of disorders and diseases, including CVDs. The pivotal step in developing an effective therapeutic strategy lies in comprehending the precise and accurate causes of diseases. Furthermore, it is imperative to determine whether autophagy serves as a cytoprotective mechanism or as a cytotoxic/cytostatic agent in the progression and prevention of diseases. To this end, it is crucial to recall that although numerous models have been developed to study the end stages of CVD, including human models, the normal healthy aging process is seldom investigated as a disease-like entity. Studies investigating cardiovascular remodeling and functions throughout the aging process as well as circulating biomarkers indicative of cardiovascular functions and risk of CVDs in aging individuals are limited, hindering understanding of the underlying causes of CVD initiation and development associated with aging. Currently, the most feasible approach is to identify the genes that undergo alterations during aging and assess whether these genes may also be involved in phenotype modulations in CVD models. To the best of our knowledge, there are no studies that systematically follow aging and monitor the state of the cardiovascular system in conjunction with omics analyses to evaluate the responses using a systems biology approach. Herein, we furnish several tables to summarize the principal knowledge and genes associated with autophagy while avoiding an extensive explanation of this mechanism since several reviews have already discussed this aspect. To sustain the central role of autophagy in CVDs, we performed a meta-analysis of different transcriptomic studies demonstrating that shared DEGs are involved in the regulation of autophagy. Considering the pervasive transcription of mammalian genomes and the importance of ncRNAs, this review describes these RNAs by also considering their potential utilization as therapeutic agents. The pleiotropic nature of miRNAs makes them difficult to use in therapy because it is important to avoid off targets in such cases. This aspect can be mitigated by using tissue-specific promoters to express miRNAs or peculiar delivery systems to target the desired cells. Another approach is to modulate genes that are ubiquitously expressed, such as in the case of Leqvio.

Most of the recently discussed ncRNAs are lncRNAs. They are important for cell functions because they can modulate gene expressions by modulating the actions of miRNAs or by interacting with DNA or transcription factors. Unfortunately, their poor conservation, which is contrary to that of miRNAs, limits the possibility of studies using different organisms and hence the knowledge of their functions and applications in therapy. Despite the challenges to the therapeutic applications of ncRNAs, they offer novel, valid, and accessible alternatives to fine-tuning autophagy. Several nucleic-acid-based therapies have been approved by the USFDA or EMA (Scalabrin and Cagnin, 2025) that are primarily based on ASOs or siRNAs. As in cancer, autophagy has a dual effect in the cardiovascular system. Enhanced autophagy in the heart can confer cardioprotective effects, but its excessive activation can be detrimental and lead to excessive degradation of intracellular components with subsequent cardiomyocyte death. In the future, ncRNAs that regulate autophagy can be utilized as substitutes for conventional drugs. Alternatively, by targeting specific ncRNAs, the sensitivity to routine drugs can be enhanced substantially, potentially enabling the reduction of their concentration and the consequent alleviation of side effects. RNAs have multifaceted applications beyond therapeutic purposes. Their expressions are altered prior to protein expressions, making them valuable diagnostic tools. NcRNAs can be secreted from cells to facilitate intercellular communication. This enables their identification through blood sampling, allowing not only diagnosis but also monitoring of pathology progression and assessing the efficacies of therapeutic interventions. In the context of substitution therapy, such as enzyme replacement therapy approved for Pompe disease (Colella, 2024), future knowledge on the mechanisms inducing reduced expressions of specific ncRNAs that are predisposed to or induce CVDs during aging may be used to undo the alterations and restore ncRNA expressions in the appropriate cells. RNAs have the advantage that they do not integrate into the genome to prevent potential tumor alterations and are stable for a certain period before being degraded; the development of therapeutic RNAs is a faster process than the development of therapeutic proteins, and these RNAs are not immunogenic if modified properly.
